# Enhancing anti-pilling performance of wool knitted fabrics *via* synergistic treatment with dopamine and silk sericin: a sustainable approach†

**DOI:** 10.1039/d5ra03257a

**Published:** 2025-07-15

**Authors:** Qi Xiao, Yuhan Wang, Wen Chen, Zhe Gao, Jing Qu, Jiajia Peng, Jiru Jia, Weifu Wang, Hafsa Jamshaid

**Affiliations:** a School of Textile Garment and Design, Suzhou University of Technology Changshu Jiangsu 215500 China xiaoqi223638@163.com; b School of Engineering and Technology, National Textile University Pakistan Hafsa@ntu.edu.pk; c School of Textile Science and Engineering, Tiangong University Tianjin 300387 China zgview@hotmail.com; d Key Laboratory of Silk Functional Materials and Technology for Textile Industry, Soochow University Suzhou Jiangsu 215123 China

## Abstract

Pilling of wool knitted fabrics during wearing or washing has long been a persistent and challenging issue. Conventionally, most anti-pilling treatments are either environmentally unfriendly or cause substantial damage to the internal structure of wool. This study adopts dopamine cross-linking silk sericin to modify wool knitted fabrics. The effects of dopamine concentration, silk sericin concentration, and cross-linking reaction time on wool fiber scale parameters (fiber friction coefficient, crimp ratio, and crimp recovery rate), anti-pilling properties, air and moisture permeability, heat retention, and mechanical properties of wool knitted fabrics were investigated. Based on the synergistic effect between dopamine and silk sericin, the wool scales were effectively encapsulated, thereby reducing the directional friction effect. Optimal conditions were identified as 1 mg mL^−1^ dopamine, 10 mg mL^−1^ silk sericin, and 24 h cross-linking reaction time. Under these conditions, a directional frictional effect value of 0.66 was achieved along with a crimp ratio of 3.41%, a recovery rate of 3.08%, resulting in a pilling grade of 5. The modified wool knitted fabrics demonstrated enhanced heat retention ratio (57.3%) and moisture permeability (9.6%), with only a marginal decrease in air permeability. The cross-linking reaction between dopamine and silk sericin exhibited a low-energy molecular thermal excitation process with an activation energy of 30.8 kJ mol^−1^.

## Abstract 2

Pilling of wool knitted fabrics during wearing or washing has long been a persistent and challenging issue.

## Graphical abstract

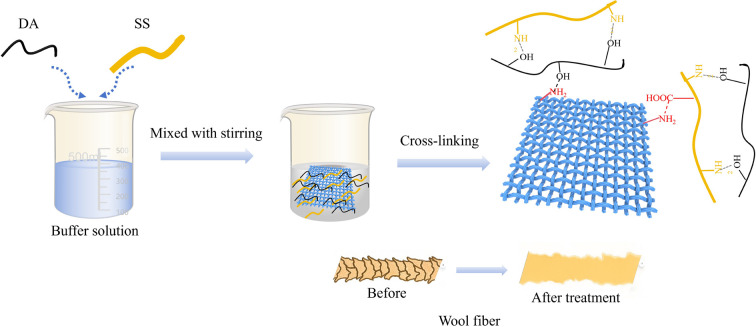

## Introduction

Wool fiber is a natural protein fiber, due to its special scale structure and crimp, which makes wool knitted fabrics have excellent elasticity, warmth, moisture absorption, and other advantages.^1,2^ However, with-scale dynamic coefficient of wool fibers is smaller than anti-scale dynamic coefficient. This difference drives wool fibers' migration toward the root during wearing or laundering. It induces the directional friction effect (DFE), promoting inter-fiber entanglement and subsequent pilling, which detrimentally impacts the aesthetic and wearing performance of wool knitted fabrics.^3,4^ The mechanism of anti-pilling treatment for wool textiles primarily targets the modification or weakening of the scale structure to mitigate DFE and reduce fiber entanglement. Current strategies to improve the anti-pilling property of wool knitted fabrics are to modify the surface of wool fibers by chemical^5^ or biological^6^ methods, which mainly include chlorination–hercosett,^7^ oxidation,^8^ reduction,^9^ plasma treatment,^10^ and enzymatic treatment.^11,12^ However, the chlorination process of removing wool fiber scales generates harmful adsorbable organic halogens (AOX), posing environmental and health risks. Enzymatic treatment alone shows limited efficacy due to the chemical resistance of wool scales, while most methods may compromise fiber strength by damaging the cortical structure. The second method is to form a thin film on the surface of wool fibers mainly by chemical methods,^13^ thus reducing the friction coefficient. However, this method impairs the handfeel and wearing comfort of wool knitted fabrics.

**Fig. 1:**
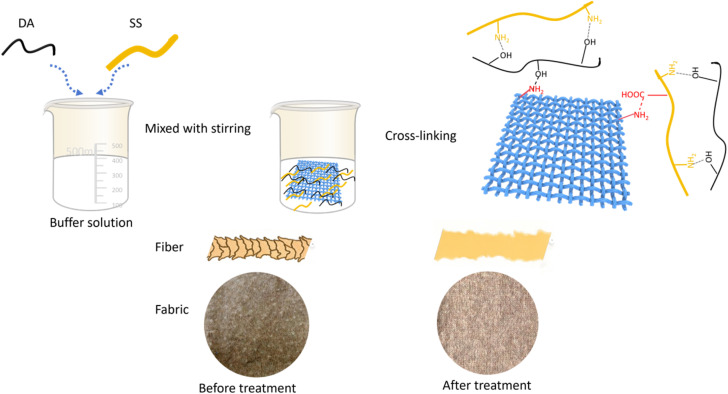
Schematic of the synergistic effects of DA and SS for anti-pilling treatment of wool fabrics.

Dopamine (DA), as a natural biomolecule, has a structure and properties similar to shellfish mucin. It is easy to oxidize and polymerize under certain conditions to generate polydopamine, which has significant adhesion ability and can adhere to the surface of almost any material, helping to promote interfacial interactions.^14,15^ Silk sericin(SS) has attracted increasing attention because of its favorable bioactivity, biocompatibility, biodegradability, and antimicrobial properties.^16^ The presence of strong side groups, such as hydroxyl and carboxyl groups, on its macromolecular chain enables it to cross-link with other polymers to form thin films. Therefore, this study proposes a synergistic approach combing DA and SS to form a cross-linked film on wool fiber surfaces, The DA/SS coating mitigates DFE by reducing frictional anisotropy, thereby improving anti-pilling property of wool knitted fabrics (Fig. 1). At the same time, dopamine helps to improve the handfeel and hygroscopicity of wool knitted fabrics.

## Experimental

### Materials and instruments

100% wool knitted fabric (Chifeng Dongli Cashmere Co., Ltd, weft flat knit, yarn density is 32 tex, gauge is 16). Dopamine hydrochloride (98%), tris(hydroxymethyl)aminomethane (standard buffer substance, ≥99.9%), silk sericin, and anhydrous sodium carbonate (≥99.9%). All the above reagents were purchased from Shanghai McLean Biochemistry Technology Co. The water used for the experiment was deionized water, which was homemade in the laboratory.

FA1104 Electronic Balance (Shanghai Shunyu Hengping Scientific Instrument Co., Ltd); HJ-6A Magnetic Stirrer (Changzhou Ronghua Instrument Manufacturing Co., Ltd); DHG-9070A Electric Blast Drying Oven (Shanghai Yihang Scientific Instrument Co., Ltd); GHH-2 Water Bath (Changzhou Gaode Instrument Manufacturing Co., Ltd); Sigma 300 Thermal Field Emission Scanning Electron Microscope (Zeiss, Germany); Nexsa X-ray photoelectron spectrometer (thermo-fisher); Nicolet 5700 Fourier Transform Infrared Spectrometer (Thermo Fisher Scientific); YG 028 Universal Tensile Tester (Ningbo Textile Instrument Factory); TU-1810SPC Ultraviolet-visible Spectrophotometer (Beijing Puxi General Instrument Co., Ltd); Y151 Fiber Friction Coefficient Instrument (Changzhou Shuanggu Dunda Mechanical and Electrical Technology Co., Ltd); YG511A Rolling Box Pilling Tester (Ningbo Textile Instrument Factory); YG362B Curl Elasticity Tester (Ningbo Textile Instrument Factory); YG461G Fully-automatic Air Permeability Tester (Ningbo Textile Instrument Factory); YG606G-II Thermal and Humidity Resistance Tester (Ningbo Textile Instrument Factory).

### Experimental methods

#### Pretreatment of wool knitted fabrics

To eliminate impurities and grease from the surface of wool knitted fabrics, the fabrics were immersed in a 5 g L^−1^ sodium carbonate solution for 10 min at room temperature. Subsequently, they were rinsed three times with deionized water and laid out flat to dry naturally.

#### Modification of wool knitted fabrics

Tris(hydroxymethyl)aminomethane (TRIS) buffer was used to prepare dopamine solutions at concentrations of 0.5, 1, 2, 5, and 10 mg mL^−1^, which were mixed with a 10 mg mL^−1^ silk sericin (SS) solution to form DA/SS blends. The mixture (pH 8.5) was dispersed at 1000 rpm. Subsequently, wool knitted fabrics were immersed in the solution at room temperature for 24 h to facilitate self-polymerization and cross-linking on wool fiber surfaces. Finally, the fabrics were taken out and dried at 60 °C for 30 min.

A 1 mg mL^−1^ DA solution was mixed with SS solutions at 6, 8, 10, 12, 14 mg mL^−1^ in TRIS buffer (pH 8.5). The DA/SS blends were dispersed at 1000 rpm. Subsequently, wool knitted fabrics were soaked in this solution under ambient conditions for 24 h, followed by drying at 60 °C for 30 min.

A 1 mg mL^−1^ DA solution and 10 mg mL^−1^ SS solution were mixed in TRIS buffer (pH 8.5), dispersed at 1000 rpm. Subsequently, wool knitted fabrics were impregnated in the solution at room temperature for 24, 36, and 48 h, respectively. Finally, these fabrics were taken out and dried at 60 °C for 30 min.

##### Preparation of dopamine and silk sericin cross-linking reaction products

DA solutions of varying concentrations were mixed with SS in TRIS buffer (pH 8.5) and dispersed at 1000 rpm. The mixed solutions were aged for 24 h to allow self-polymerization and cross-linking reactions.

#### Performance testing and characterization

##### Fiber morphological characterization

A Sigma 300 thermal field emission scanning electron microscope (SEM) was used to observe the morphological characteristics of wool fiber surfaces, with a test voltage of 5 kV and gold sputtering before testing. Energy dispersive X-ray spectroscopy (EDS) was conducted in conjunction with SEM to investigate variations in the elemental composition of the wool fibers.

##### Fabric surface functional group analysis

A Nicolet 5700 Fourier transform infrared spectroscopy (FTIR) spectrometer was used to observe changes in the functional groups on the fabric surface. X-ray photoelectron spectroscopy (XPS) analysis was performed by using a Nexsa XPS spectrometer.

##### Anti-pilling properties of fabrics

In accordance with ISO 12945-1: 2000 standard “Textiles – Determination of fabric propensity to surface fuzzing and to pilling – Part 1: Pilling box method”, a YG511A Rolling Box Pilling Tester was employed to assess the anti-pilling properties of wool knitted fabrics. The light box used for rating is a standard light source box.

##### Fabric mechanical properties

In accordance with ISO 3303: 1990, “Textiles—Determination of bursting strength—Steel ball method”, YG028 universal tensile tester was used to assess the strength of wool knitted fabrics.

##### Fiber friction properties

In accordance with GB/T 45179-2024, “Determination of the coefficient of friction of chemical fiber staple fibers – drum method”, a Y151 fiber friction coefficient tester was utilized to evaluate the friction coefficients of wool fibers.

##### Fiber crimp properties

According to GB/T 14338-2022, “Man-made fibre—Test method for crimping performance of staple fibre”, a YG362B crimp tester was used to measure the crimp properties of wool fibers.

##### Air and moisture permeability of fabrics

According to ISO 9237: 1995, “Textiles – Determination of the permeability of fabrics to air”, a YG461G automatic air permeability meter was used to test the air permeability of wool knitted fabrics before and after treatment. According to ISO 15496, “Textiles – measurement of water vapour permeability of textiles for the purpose of quality control”, a YG601H-III computerized fabric moisture permeability tester was used to test the moisture permeability of wool knitted fabrics.

##### Heat retention properties of fabrics

According to ISO 11092: 1993, “Textiles-Physiological effects-Measurement of thermal and water-vapour resistance under steady-state conditions(sweating guarded-hotplate test)”, the heat retention properties of wool knitted fabrics were tested by using a YG606G-II thermal resistance and moisture resistance tester.

##### Kinetic analysis of DA/SS crosslinking

An appropriate amount of dopamine and silk sericin cross-linking reaction product was collected and analyzed using a UV-Visible spectrophotometer^17^ within the wavelength range of 200–800 nm.^1^ The dopamine reactant consumption was plotted against time, with the slope of this curve representing the reaction rate. The reaction rates at various initial concentrations were determined, and the reaction rate equation (eqn (1)) was utilized to calculate the value of *k* through linear fitting or numerical methods. The aforementioned experiment was repeated at different temperatures to obtain the reaction rate constants (*k*). According to the Arrhenius equation (eqn (2)), a plot of ln *k versus* 1/*T* yielded a straight line, which was characterized as *−E*_a_/*R*, and from this, the activation energy, *E*_a,_ was calculated.

1 *r* = *k*[*A*][*B*]

where *r* is the reaction rate, g (L^−1^ min^−1^); *k* is the reaction rate constant, L (g^−1^ min^−1^); and [*A*] and [*B*] are the concentrations of dopamine and silk sericin, g L^−1^, respectively.

2 *k* = *me*^−*E*_a_/*RT*^

where *m* is the prefactor; *E*_a_ is the activation energy, kJ mol^−1^; *R* is the gas constant, which takes the value of 8.314 × 10^−4^ kJ (mol^−1^ K^−1^); and *T* is the absolute temperature, *K*.

## Results and discussion

### Analysis of functional groups of wool fibers

**Fig. 2:**
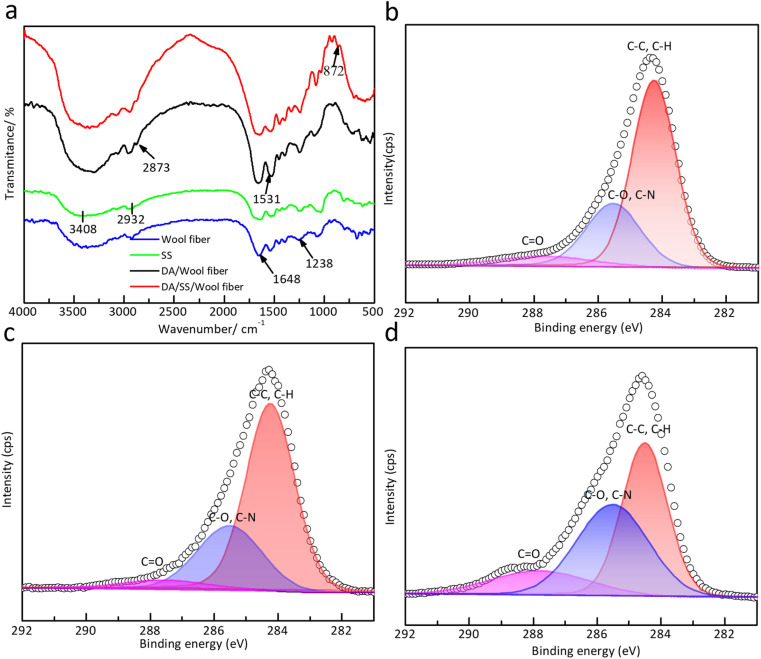
(a) Infrared spectra of wool fibers before and after treatment; (b)–(d) XPS spectra of C 1s from wool fiber, DA/wool fiber, and DA/SS/wool fiber.

As depicted in Fig. 2(a), the characteristic peaks of wool fibers, DA/wool fibers, and DA/SS/wool fibers exhibit remarkable similarity. The absorption bands between 3500 and 2800 cm^−1^ are related to the stretching vibrations of O–H, C–H, and N–H groups. Notably, the peaks at 1648 cm^−1^ (amide I) and 1238 cm^−1^ (amide II) indicate that the original backbone structure of silk sericin remains intact, suggesting no significant structural alteration during treatment. The absorption bands at 2873 cm^−1^, 1531 cm^−1^, and 872 cm^−1^ correspond to the functional groups –CH_2_, –NH, and –CN in the polydopamine (DA) molecular structure, respectively. The characteristic peak of the dopamine cross-linked silk sericin, around about 872 cm^−1^, arises from the out-of-plane bending vibrations of aromatic C–H bonds in the DA structure. Meanwhile, a stretching vibration peak of the quinone group emerges at ∼1750 cm^−1^, evidencing the oxidation of DA's catechol groups to quinone groups. This observation confirms the self-polymerization of DA on the wool fiber surfaces. XPS was performed to characterize the chemical structures of wool fibers. The C 1s spectra of pristine wool fibers, DA/wool fibers, and DA/SS/wool fibers were illustrated in Fig. 2(b)–(d). Three distinct peaks at 284.5 eV, 285.5 eV, and 288 eV were responsible for C–C/C–H, O–C–O/C=O, and C=N, respectively. The C–C/C–H components derive from fatty acids and peptide chains on the wool fiber surface, while O–C–O/C=O and C=N are characteristic peaks of DA and sericin. Compared to pristine wool fibers, the treated fibers exhibit an increased peak area of C–N bonds, indicating the successful adsorption of DA and sericin onto the wool fiber surface. The decrease in C–C/C–H contents in the treated fibers is presumably attributed to the encapsulation of wool scales by the sericin protein film formed during the treatment process.

### Effect of dopamine concentration on anti-pilling properties of wool knitted fabrics

**Fig. 3:**
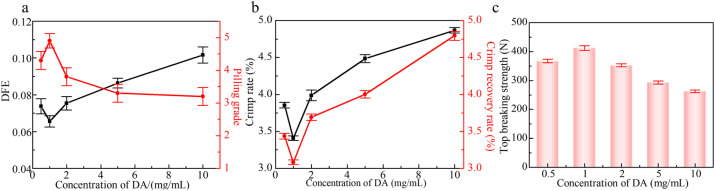
(a) Effect of DA concentration on DFE of wool fibers and the pilling grade of wool knitted fabrics; (b) effect of DA concentration on the crimping properties of wool fibers; (c) effect of DA concentration on the top breaking strength of wool knitted fabrics.

**Fig. 4:**
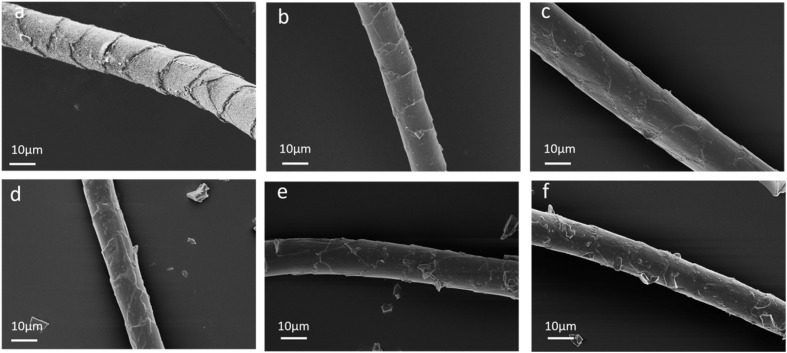
Surface morphology of wool fibers after treatment with different dopamine concentrations: (a) untreated wool fiber; (b) 0.5 mg mL^−1^; (c) 1 mg mL^−1^; (d) 2 mg mL^−1^; (e) 5 mg mL^−1^; (f) 10 mg mL^−1^.

*u*_ad_ is the anti-scale dynamic coefficient, *u*_wd_ is the with-scale dynamic coefficient, and the differential friction effect (DFE) is equal to *u*_ad_ − *u*_wd_. DFE can be used to evaluate the anti-pilling property. The smaller its value is, the better the anti-pilling property is.^18,19^Fig. 3(a) depicts the effect of dopamine concentration on the DFE of wool fibers and the anti-pilling properties of wool knitted fabrics. From Fig. 3(a), it can be illustrated that with the increase of dopamine concentration, DFE shows a trend of decreasing and then increasing, while the pilling grade of wool knitted fabrics shows a converse trend (increasing and then decreasing). The minimum DFE (0.66) and highest pilling grade (grade 5) occur at a dopamine concentration of 1 mg mL^−1^. This is attributed to the formation of a polydopamine coating *via* dopamine self-polymerization on wool fibers, which enhances the grafting reaction between silk sericin and wool. The coating encapsulates surface scales, as evidenced by SEM observations (Fig. 4(a)–(f)). It can be seen that with the increase of dopamine concentration, the scales on the surface of wool fibers are gradually encapsulated, and the surface is the smoothest when the dopamine concentration is 1 mg mL^−1^. By further increasing the dopamine concentration, the polymerization reaction of dopamine on the fiber surface generates excessive polydopamine. It results in agglomerating together, which makes the surface of the fibers rough and increases DFE.

The crimp of wool fibers not only provides the holding force during fiber friction, but also affects the locking knot during fiber friction, *i.e.*, the smaller the crimp rate and crimp recovery rate are, the less prone to pilling of wool fibers are.^20^Fig. 3(b) illustrates the effect of dopamine concentration on the crimp properties of wool fibers. It can be seen that with the increase of dopamine concentration, the crimp ratio and crimp recovery rate of wool fibers exhibit a decreasing and then increasing trend. When the concentration of dopamine is 1 mg mL^−1^, the crimp ratio and crimp recovery rate of wool fibers are the smallest, which are 3.41% and 3.08%, respectively. This is because the initial increase in dopamine concentration forms a polymer film on the wool fibers. It has a certain degree of rigidity, limiting the crimp properties of wool. And the fiber surface becomes smoother, which reduces inter-fiber friction and the locking effect. Therefore, the crimp rate and the crimp recovery rate of wool fibers are decreased. With the further increase of dopamine concentration, the film on the fiber surface is thickened, as shown in Fig. 4(d)–(f). The polydopamine layer becomes pliable and could adapt to the crimp deformation of the wool fibers, which leads to a rebound in the crimp rate and the crimp recovery rate of the fibers. In addition, the high concentration of dopamine leads to an excessive cross-linking reaction between the fibers and the polymer. This results in uneven crimp distribution of the fibers. This is evidenced by the enlarged error bars in Fig. 3(b). Therefore, the fibers are more likely to break at the weak point when subjected to friction by an external force. Fig. 3(c) illustrates the effect of dopamine concentration on the top breaking strength of wool knitted fabrics, which further proves that high dopamine concentration leads to easy brittle breakage of some of the fibers and a decrease in the top breaking strength of the fabrics.

### Effect of silk sericin concentration on the anti-pilling properties of wool knitted fabrics

**Fig. 5:**
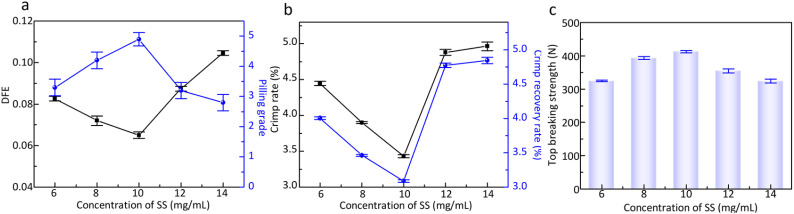
(a) Effect of SS concentration on DFE of wool fibers and the pilling grade of wool knitted fabrics; (b) effect of SS concentration on the crimp properties of wool fibers; (c) effect of SS concentration on the top breaking strength of wool knitted fabrics.

**Fig. 6:**
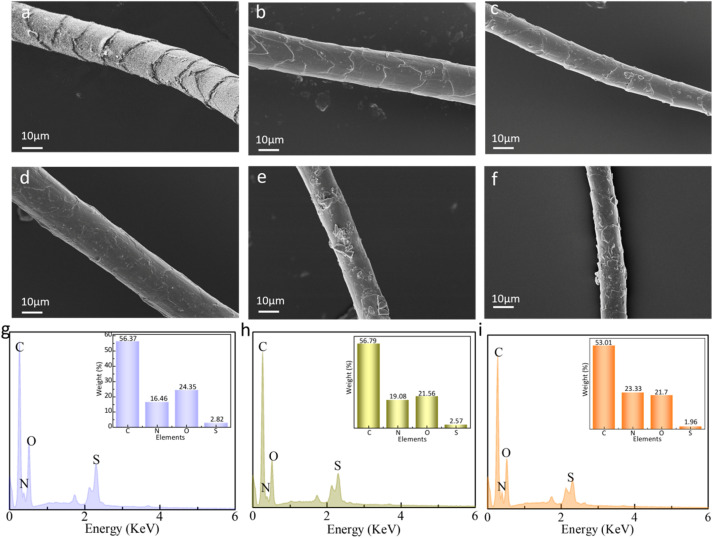
SEM images and EDS spectra of wool fibers at different SS concentrations: (a) untreated wool fiber; (b) 6 mg mL^−1^; (c) 8 mg mL^−1^; (d) 10 mg mL^−1^; (e) 12 mg mL^−1^; (f) 14 mg mL^−1^; (g) EDS of wool fiber; (h) EDS of DA/wool fiber; (i) EDS of DA/SS/wool fiber.

Fig. 5(a) illustrates the influence of SS concentration on DFE of wool fiber and the anti-pilling property of wool knitted fabrics. From Fig. 5(a), it can be seen that the DFE of wool fibers shows a trend of decreasing and then increasing with the increase of the concentration of silk sericin, while the pilling grade of wool knitted fabrics firstly increases and then decreases. The pilling grade of wool knitted fabrics is grade 5 at an SS concentration of 10 mg mL^−1^. This is because when SS concentration increases from low concentration to a certain degree, a more continuous film is formed on the wool fiber surface, as shown in Fig. 6(a)–(d). It can be seen that the film has a certain covering and lubricating effect on the fiber scales. So, the difference between the *u*_ad_ and *u*_wd_ decreases, which leads to DFE decrease. From Fig. 6(e) and (f), it can be indicated that excessive SS concentration causes surface accumulation of SS. The roughness of the fiber surface is increased, which leads to an increase in the DFE.

Fig. 5(b) depicts the effect of SS concentration on the crimp rate and crimp recovery rate of wool fibers. With the increase of SS concentration, the wool fiber crimp rate and crimp recovery rate show a decreasing and then increasing trend. The initial increase of SS concentration forms a more continuous interfacial layer on the wool fiber surface, restricting the mobility of keratin molecular chains and reducing fiber crimping under external forces. At the same time, SS molecules compete with keratin for hydrogen bonding, disrupting the native intermolecular interactions that maintain fiber crimp structure, thereby decreasing crimp ratio and crimp recovery rate. When SS concentration is further increased, a thicker SS film develops, enhancing intermolecular interaction between SS and keratin while providing additional elastic support to wool fibers. This leads to an increase in the crimp ratio and the crimp recovery rate. However, excessive SS induces uncontrolled cross-linking between dopamine, SS, and the wool fibers, causing heterogeneous crimp distribution. Fig. 5(c) shows that high SS concentrations reduce the top breaking strength of wool knitted fabrics, as stress concentrates at weak points induced by non-uniform cross-linking. As illustrated in Fig. 5(d)–(f), EDS analysis confirmed that nitrogen content on the wool fiber surface increased while sulfur content decreased. This indicated that the wool fiber scales were enveloped by the DA/SS film.

### Effect of cross-linking reaction time on anti-pilling properties of wool knitted fabrics

**Fig. 7:**
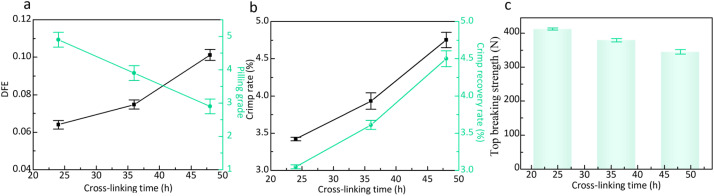
(a) Effect of cross-linking reaction time on DFE of wool fibers and the pilling grade of wool knitted fabrics; (b) effect of cross-linking reaction time on the crimp properties of wool fibers; (c) effect of cross-linking reaction time on the top-breaking strength of wool knitted fabrics.

**Fig. 8:**
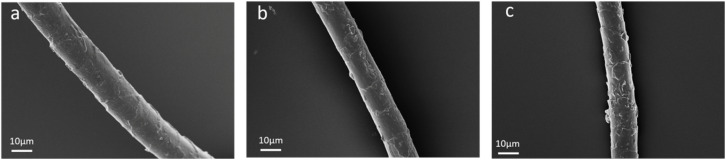
Characteristics of the surface morphology of wool fibers with different cross-linking reaction times: (a) 24 h; (b) 36 h; (c) 48 h.

Fig. 7(a) shows the effect of cross-linking reaction time on DFE of wool fibers and pilling grade of wool knitted fabrics. It can be indicated that DFE gradually increases with the increase of cross-linking reaction time, while the pilling grade gradually decreases. This is because when the cross-linking reaction time is 24 h, the cross-linking of DA and SS occurs more evenly on the wool fiber surface. Fig. 8(a) shows the surface morphology of wool fibers under different cross-linking reaction times. It can be seen that when the crosslinking reaction time is 24 h, a layer of more uniform and smooth polymer coating is formed on the fiber surface, reducing *u*_ad_ and *u*_wd_ of wool fibers. Therefore, the DFE is in a more desirable range. The fibers are not easy to entangle with each other when subjected to external friction, reducing the possibility of pilling ball formation. Prolonged cross-linking reaction time leads to excessive cross-linking, as shown in Fig. 8(b) and (c). It can be seen that a thicker and uneven coating is formed, increasing the roughness of the fiber surface and the DFE. In the event of external forces, the fibers are easier to entangle with each other to form pilling balls. Therefore, the anti-pilling performance of wool knitted fabrics decreases.

The effect of cross-linking reaction time on the crimp properties of wool fibers is illustrated in Fig. 7(b). It can be seen that the crimp rate and crimp recovery rate of wool fibers gradually increased with the increase of cross-linking reaction time. This is because the crimp ratio and crimp recovery rate of wool fibers obtained at cross-linking reaction time of 24 h are in an ideal state, with the values of 3.42% and 3.05%, respectively. When fibers are subjected to an external force, they can quickly recover the crimp state and maintain the close arrangement between the fibers, thus enhancing the anti-pilling properties of the fabrics. With the prolongation of the cross-linking reaction time, excessive cross-linking may occur, destroying the original internal structure of wool fibers. From Fig. 7(b), it can be seen that the error bars of the crimp ratio and crimp recovery rate of wool fibers at 36 h and 48 h of cross-linking reaction time are larger than those at 24 h. This indicates that some regions of wool fibers are over-curled and some regions are under-curled. When the fibers are subjected to the friction of the external force, the over-curled regions are prone to stress concentration. It leads to fiber breakage or extraction. On the other hand, the under-curled region does not provide sufficient holding force to stop the movement of the fibers, thus making the fabric more prone to pilling. Fig. 7(c) shows a gradual decrease in top breaking strength with prolonged cross-linking. Excessive reaction time causes fiber brittleness due to over-cross-linking, creating weak points that fail preferentially under tensile stress, consistent with the morphological observations (as shown in Fig. 8).

### Wearing performance of wool knitted fabrics after treatment

**Fig. 9:**
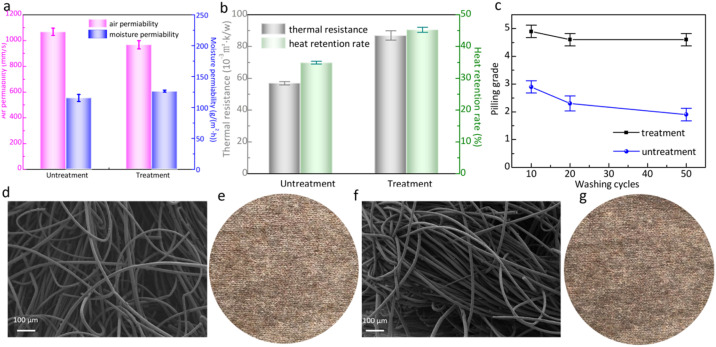
Wearing performance of wool knitted fabrics before and after treatment: (a) air and moisture permeability; (b) thermal resistance and heat retention rate; (c) anti-pilling durability of wool knitted fabrics; (d) and (e) SEM image and physical image of wool fabric after 20 washing cycles; (f) and (g) SEM image and physical image of wool fabric after 50 washing cycles.

To investigate whether the wearing properties of wool knitted fabrics are affected after treatment, the wearing performance of wool fabrics is tested. Fig. 9(a)–(c) shows the changes in air permeability, moisture permeability, and heat retention of wool knitted fabrics after treatment. From Fig. 9(a), it can be seen that the air permeability of wool knitted fabrics decreases slightly, which is attributed to the polymerization and cross-linking reactions of DA and SS occurring on the wool fiber's surface to form a thin film. This leads to a slight increase in the fiber diameter, reducing inter-fiber pore space, and impeding air flow. Conversely, the moisture permeability of the wool knitted fabric is improved due to the hydrophilic groups (amino, hydroxyl, and carboxyl) in SS molecules. This groups adsorb water vapor at high air pressure and transfer it to the other side of the fabric, enhancing air permeability. From Fig. 9(b), heat retention capacity increases as the cross-linked DA-SS film strengthens interfacial adhesion between wool fibers. The film acts as a thermal barrier, reducing direct air-fiber contact and minimizing heat dissipation.

### Anti-pilling durability of modified wool knitted fabrics

The optimal process parameters are determined as 1 mg mL^−1^ DA, 10 mg mL^−1^ SS, and 24 h cross-linking time. The pilling grade of the wool knitted fabrics is 5 by using the optimized process parameters. To evaluate the anti-pilling durability of the modified wool knitted fabrics, the wool knitted fabrics are subjected to 10, 20, and 50 washing styles according to ISO 6330: 2012 “Textiles—Domestic washing and drying procedures for textile testing”, and then tested for pilling property. The results are shown in Fig. 9(c). It can be seen that the pilling grade of the untreated wool knitted fabric is 2 after 50 washing cycles. After 20 and 50 washing styles of the modified wool knitted fabrics, the pilling grade is still 4.5. SEM images and physical images of wool fabrics after 20 and 50 washing cycles can be illustrated in Fig. 9(d)–(g), which could be indicated that the films were still wrapped around the surface of the wool fibers, and scales can hardly be seen. Therefore, the modified wool knitted fabrics have better anti-pilling durability.

### Mechanism of the cross-linking reaction of dopamine with silk sericin

**Fig. 10:**
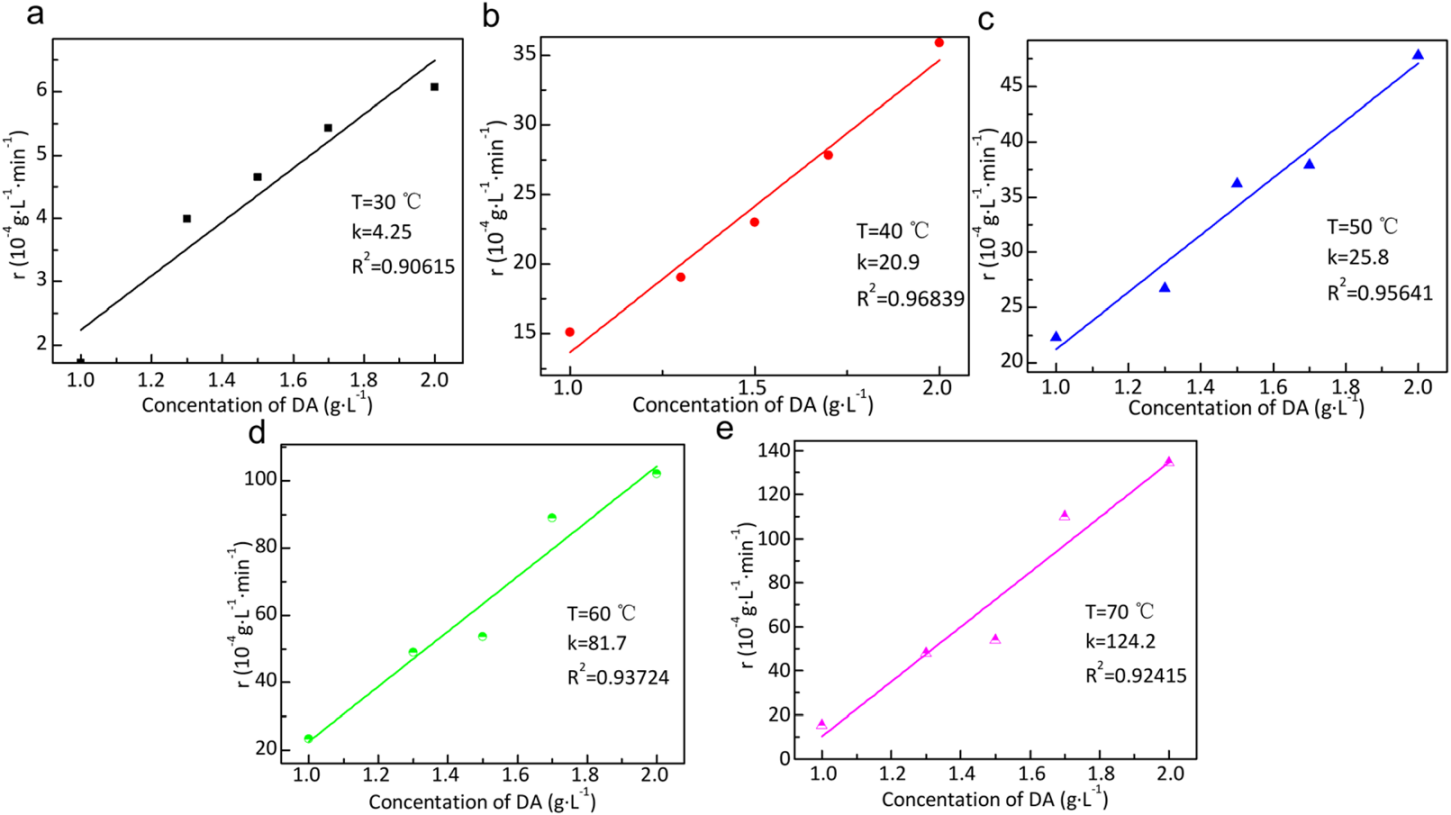
Fitted curves for the reaction rate constant of the cross-linking of DA with SS at different reaction temperatures: (a) 30 °C; (b) 40 °C; (c) 50 °C; (d) 60 °C; (e) 70 °C.

In order to further investigate the reaction mechanism of DA and SS cross-linking, UV spectroscopy analysis is adopted to collect the relevant kinetic parameters and determine the reaction stages and reaction processes. Fig. 10 shows the fitted curves of the reaction rate constants at different reaction temperatures. It can be seen that the cross-linking reaction rate constants show an obvious temperature dependence with the change of reaction temperature. The fitted curves of the reaction rate constants in the range of 30–70 °C showed a clear linear relationship. The reaction rate constants are 4.25, 20.9, 25.8, 813.7, and 124.2 L (g^−1^ min^−1^), respectively. Therefore, linear fitting of the reaction rate constants and temperature according to the Arrhenius equation gives a calculated activation energy of 30.8 kJ mol^−1^. This indicates that the cross-linking reaction is a typical molecularly thermally-excited reaction, which is characterized by a relatively easy reaction to carry out with a low activation energy. Therefore, dopamine oxidizes under alkaline conditions to form *o*-quinone. Silk sericin, which contains functional groups such as amino and hydroxyl groups, undergoes a nucleophilic addition reaction with *o*-quinone to form a covalent bond cross-linking. This reaction improves the scale parameters of wool fibers, such as friction coefficient, crimp rate, and crimp recovery rate, and thus improves the anti-pilling properties of wool knitted fabrics.

## Conclusion

The study revealed that the cross-linking reaction between dopamine and silk sericin effectively improved the anti-pilling properties of wool knitted fabrics. The optimization of dopamine concentration, silk sericin concentration, and cross-linking reaction time was crucial for balancing fiber properties and fabric performance. Excessive concentrations of either component resulted in increased directional frictional effect, crimp ratio, and crimp recovery rate, leading to non-uniform crimp patterns and enhanced susceptibility to pilling. Under optimal conditions (1 mg mL^−1^ dopamine, 10 mg mL^−1^ silk sericin, 24 h reaction time), the wool fibers exhibited a directional frictional effect of 0.66, crimp ratio of 3.41%, and crimp recovery rate of 3.08%, yielding a pilling grade of 5. Additionally, the heat retention rate of the wool knitted fabric was improved by 57.3%, moisture permeability by 9.6%, with only a slight decrease in air permeability. The low activation energy of 30.8 kJ mol^−1^ for the covalent bond cross-linking reaction facilitated its efficient execution, demonstrating the potential of this eco-friendly approach for enhancing the durability and comfort of wool knitted fabrics. In summary, this method using dopamine and silk sericin provides an alternative for eco-friendly anti-pilling treatment of wool fabrics, demonstrating the promising commercial application.

## Authors contribution

Qi Xiao: writing – original draft, validation, methodology, investigation, conceptualization, funding acquisition. Yuhan Wang: methodology, validation, investigation, resources. Wen Chen: methodology, validation, investigation. Zhe Gao and Jing Qu: supervision, methodology, formal analysis. Jiru Jia and Jiajia Peng: conceptualization, validation, resources, methodology. Hafsa Jamshaid and Weifu Wang: methodology, supervision, writing – review & editing.

## Conflicts of interest

The authors declare that they have no known competing financial interests or personal relationships that could have appeared to influence the work reported in this paper.

## Supplementary Material

RA-015-D5RA03257A-s001

## Data Availability

The data supporting this article have been included as part of the ESI.†.
