# Supplementary material for: Enhancing anti-pilling performance of wool knitted fabrics via synergistic treatment with dopamine and silk sericin: a sustainable approach
Source: RSC Adv. 2025 Jul 15;15(30):24862–71. doi: 10.1039/d5ra03257a (PMC12260831; doi:10.1039/d5ra03257a)
Supplement: RA-015-D5RA03257A-s001 [file RA-015-D5RA03257A-s001.pdf]

Supplementary Information (SI) for RSC Advances.
This journal is © The Royal Society of Chemistry 2025

Table 1 FTIR data

Wavenumber	Wool fiber	SS	DA/Wool fiber	DA/SS/Wool fiber
499.4729	63.5418	44.98734	86.43869	88.50951
501.4014	63.63546	44.5065	86.5148	88.38752
503.3298	63.55659	43.68824	86.58316	88.2785
505.2583	63.50316	42.40804	86.64375	88.18245
507.1868	63.42365	40.66982	86.69658	88.09936
509.1152	62.50445	39.54359	86.74166	88.02924
511.0437	64.59203	38.07738	86.77897	87.97209
512.9722	66.05624	37.97644	86.80853	87.92791
514.9006	66.5446	38.62706	86.83033	87.89669
516.8291	66.16751	38.54225	86.84436	87.87844
518.7576	64.95356	38.97615	86.85064	87.87316
520.686	63.97258	38.59239	86.71139	88.01813
522.6145	63.56268	37.89104	86.6351	88.16796
524.543	62.99735	37.54737	86.68806	88.3925
526.4714	62.04179	37.38697	86.84211	88.72903
528.3999	60.93573	37.30849	86.9373	88.96327
530.3284	59.90828	37.13589	86.95836	88.96624
532.2568	59.3056	37.08348	86.95727	88.78608
534.1853	58.94419	36.86758	87.01312	88.64369
536.1138	58.62861	36.50494	87.04365	88.35862
538.0422	57.96037	36.01054	87.00401	87.6139
539.9707	57.06133	35.58365	87.02815	86.60237
541.8992	56.87183	35.11353	87.23531	85.81723
543.8276	57.02459	34.83194	87.60465	85.4735
545.7561	57.55086	34.92015	87.93546	85.35621
547.6846	57.84242	35.01244	88.16411	85.12017
549.613	57.75992	35.04827	88.30495	84.66251
551.5415	57.8215	35.07812	88.4229	84.41732
553.47	58.28323	35.08787	88.45533	84.49153
555.3984	58.52518	35.0734	88.31626	84.55686
557.3269	58.68088	35.21627	88.03561	84.40511
559.2554	58.88062	35.25652	87.70137	84.23392
561.1838	59.03924	35.19425	87.45018	84.18612
563.1123	59.09748	35.29768	87.30741	84.19992
565.0408	59.30008	35.20576	87.14879	84.22598
566.9692	60.4958	35.03926	86.83737	84.32474
568.8977	61.35281	34.91619	86.47913	84.70888
570.8262	61.26158	34.88777	86.24633	85.27188
572.7546	60.96024	34.97276	86.13224	85.61092
574.6831	61.15041	35.1791	86.01929	85.64295
576.6116	61.7531	35.40952	85.89525	85.53222

578.54	62.42786	35.40958	85.8433	85.38051
580.4685	63.00484	34.89292	85.89364	85.28213
582.397	63.24068	34.56064	86.00528	85.43965
584.3254	62.80025	34.36057	86.1328	85.66087
586.2539	62.18844	34.15093	86.2798	85.56005
588.1824	61.97181	33.95737	86.45413	85.32919
590.1108	61.76621	33.84432	86.58941	85.40849
592.0393	61.54869	33.49997	86.59471	85.71407
593.9678	61.24504	33.137	86.44874	86.06445
595.8962	61.06944	33.22353	86.22236	86.35563
597.8247	60.95896	33.72932	85.96587	86.4927
599.7532	60.98676	34.12746	85.76666	86.43727
601.6816	60.48011	34.00758	85.67224	86.4499
603.6101	60.02245	33.98609	85.57419	86.65928
605.5386	59.65556	34.07494	85.36572	86.93423
607.467	59.33905	34.19951	85.08191	86.95373
609.3955	59.53393	34.51936	84.86761	86.54425
611.324	60.35573	34.53122	84.73684	85.90208
613.2524	60.94324	34.3301	84.611	85.35428
615.1809	60.82401	34.05799	84.43962	84.96987
617.1094	60.46269	34.10558	84.30145	84.54019
619.0378	60.10421	34.58329	84.34103	84.06594
620.9663	59.86767	34.97502	84.56942	83.62169
622.8948	59.70123	34.92066	84.88605	83.25337
624.8232	59.56654	34.62849	85.2242	82.96928
626.7517	59.51146	34.56237	85.57875	83.03897
628.6802	59.68936	34.90446	85.92882	83.53181
630.6086	59.83361	34.97248	86.26608	84.08595
632.5371	60.43368	34.69262	86.57332	84.48141
634.4656	61.76688	34.55105	86.75079	84.77457
636.394	62.89385	34.58755	86.8093	85.08188
638.3225	63.75442	34.75063	86.86569	85.24616
640.251	64.59617	35.29149	86.98189	85.30893
642.1794	65.22308	35.84742	87.07661	85.35279
644.1079	65.76855	36.12606	87.10032	85.40084
646.0364	66.44583	36.13742	87.09029	85.44184
647.9648	67.28283	35.99529	87.12856	85.52388
649.8933	68.05299	35.85771	87.20185	85.61255
651.8218	68.80213	35.60871	87.23348	85.5329
653.7502	69.522	35.4786	87.18951	85.27459
655.6787	69.83668	35.71323	87.09797	84.7096
657.6072	69.70467	35.73141	87.00635	83.84532
659.5356	69.31306	35.64235	86.95422	82.97553

661.4641	68.9932	35.62081	86.94232	82.41773
663.3926	68.81866	36.02748	86.94651	81.97454
665.321	68.64493	36.54829	86.92336	81.4007
667.2495	68.31292	36.66648	86.88682	80.74009
669.178	68.17432	37.0079	86.86797	80.20673
671.1064	68.1637	37.77231	86.8556	79.86326
673.0349	68.02601	38.44854	86.79444	79.69552
674.9634	68.19318	38.81093	86.78277	79.62558
676.8918	68.11256	38.86001	86.8753	79.66901
678.8203	68.02489	38.69928	86.99373	79.98077
680.7488	68.19531	38.59876	87.06225	80.64416
682.6772	68.11261	38.62139	87.09161	81.52896
684.6057	68.02111	38.68207	87.083	82.39947
686.5342	67.96975	38.6337	87.02479	83.18219
688.4626	67.84269	38.45212	86.92013	83.87008
690.3911	67.97055	38.32435	86.82502	84.45751
692.3196	67.96386	38.10556	86.78222	84.94114
694.248	67.77328	37.61764	86.82662	85.32129
696.1765	67.48044	36.89247	86.92501	85.67074
698.105	67.15278	36.48525	87.01583	86.02651
700.0334	66.56663	36.45478	87.10381	86.30763
701.9619	65.93913	36.62635	87.23375	86.4934
703.8904	65.63751	36.75997	87.42406	86.70088
705.8188	65.57199	36.78102	87.6557	86.97879
707.7473	65.84238	36.7607	87.97727	87.17072
709.6758	66.26502	37.09035	88.40275	87.18387
711.6042	66.40608	37.66967	88.83162	87.13705
713.5327	66.42762	38.22001	89.17668	87.09066
715.4612	66.52782	38.98618	89.40058	87.0034
717.3896	66.33401	39.65675	89.48177	86.78299
719.3181	66.00768	40.15521	89.48049	86.47007
721.2466	65.78345	40.62934	89.47876	86.20056
723.175	65.48565	41.07142	89.49487	86.12463
725.1035	65.50081	41.52259	89.48038	86.23754
727.032	65.98447	41.98837	89.43613	86.4416
728.9604	66.74248	42.39127	89.40815	86.66971
730.8889	67.1608	42.7703	89.44326	86.87301
732.8174	67.0003	43.14992	89.51615	87.00271
734.7458	66.99144	43.55306	89.51924	87.0643
736.6743	67.28943	44.02305	89.43743	87.14771
738.6028	67.47282	44.35003	89.40961	87.4536
740.5313	67.60397	44.56265	89.48593	88.12135
742.4597	67.96897	44.7495	89.62206	88.774

744.3882	68.17373	45.16706	89.80598	89.00702
746.3167	68.21449	45.71191	90.04599	88.93638
748.2451	68.43132	46.27996	90.30484	89.03866
750.1736	68.70496	46.89806	90.5342	89.36233
752.1021	68.98674	47.42578	90.70947	89.53629
754.0305	69.43051	47.97063	90.81489	89.38464
755.959	69.9251	48.73566	90.89843	89.13017
757.8875	70.52024	49.49261	91.05782	89.05448
759.8159	71.13345	50.16074	91.25946	89.23199
761.7444	71.41547	50.81495	91.38581	89.62003
763.6729	71.57294	51.44465	91.37073	90.03687
765.6013	71.86113	52.00354	91.27419	90.26234
767.5298	72.1923	52.54474	91.22605	90.29134
769.4583	72.74097	53.17621	91.2817	90.1874
771.3867	73.47555	53.87601	91.3647	89.98434
773.3152	74.09055	54.36167	91.353	89.75265
775.2437	74.68056	54.83217	91.25749	89.70468
777.1721	74.99906	55.70413	91.12854	89.9159
779.1006	75.06654	56.75948	91.01941	90.12868
781.0291	75.34328	57.75009	90.95363	89.99602
782.9575	75.44276	58.69534	90.89135	89.6509
784.886	75.17355	59.6148	90.75554	89.50492
786.8145	74.82435	60.50826	90.56728	89.61883
788.7429	74.6142	61.37318	90.37216	89.60288
790.6714	74.57715	62.33128	90.17168	89.20168
792.5999	74.68752	63.34422	89.95581	88.61867
794.5283	74.67497	64.40473	89.73596	88.31305
796.4568	74.64851	65.52247	89.52686	88.42099
798.3853	74.91623	66.57871	89.32447	88.67424
800.3137	75.31863	67.54423	89.16341	88.92106
802.2422	75.58559	68.54578	89.08161	89.17552
804.1707	75.76743	69.52444	89.08481	89.40317
806.0991	75.9834	70.42568	89.14927	89.61212
808.0276	76.4359	71.13095	89.22913	89.82383
809.9561	76.96444	71.7491	89.30048	90.13394
811.8845	77.45886	72.45612	89.40764	90.49532
813.813	78.28686	73.36639	89.66335	90.92199
815.7415	79.36027	74.46217	90.06344	91.37934
817.6699	80.32455	75.45459	90.52519	91.85374
819.5984	81.1584	76.21887	90.98998	92.30226
821.5269	82.00788	77.13186	91.45916	92.66547
823.4553	82.7137	78.42979	91.94917	92.94671
825.3838	83.0089	79.65436	92.49931	93.09616

827.3123	83.44099	80.52139	93.11675	93.08798
829.2407	83.87357	81.34576	93.73173	93.03399
831.1692	84.23122	82.31178	94.28101	93.15793
833.0977	84.735	83.04534	94.76394	93.48077
835.0261	85.23151	83.55257	95.2558	93.71888
836.9546	85.62106	84.09656	95.78883	93.82792
838.8831	85.91875	84.5155	96.3134	94.07576
840.8115	86.32475	84.97912	96.72769	94.47146
842.74	86.95849	85.45979	96.94739	94.73083
844.6685	87.56303	85.82832	96.974	94.82017
846.5969	87.92155	86.04816	96.92528	94.93286
848.5254	88.25396	86.10946	96.94212	95.09424
850.4539	88.71557	86.10719	97.06095	95.10515
852.3823	89.15638	85.97742	97.21333	95.0195
854.3108	89.44398	85.77245	97.28501	95.0504
856.2393	89.52293	85.65766	97.26229	95.22648
858.1677	89.79713	85.5492	97.2308	95.37801
860.0962	90.40605	85.46658	97.26018	95.40337
862.0247	90.84884	85.5864	97.34087	95.41162
863.9531	91.10679	85.44521	97.4623	95.48542
865.8816	91.24414	85.17239	97.59923	95.61325
867.8101	91.30785	85.28867	97.68898	95.72864
869.7385	91.51758	85.72188	97.78422	95.72785
871.667	91.92712	86.06278	97.96397	95.62994
873.5955	92.4885	86.39561	98.24238	95.57261
875.5239	92.84982	87.01276	98.51286	95.72458
877.4524	93.15443	87.69604	98.70041	95.98634
879.3809	93.44823	88.32384	98.78166	96.184
881.3093	93.64809	89.01909	98.77185	96.3356
883.2378	93.75642	89.81152	98.72357	96.53865
885.1663	94.07361	90.64828	98.70003	96.85183
887.0947	94.45148	91.71566	98.74633	97.18037
889.0232	94.59399	92.7535	98.82006	97.40085
890.9517	94.55465	93.58522	98.86692	97.47405
892.8801	94.4565	94.14075	98.88705	97.53582
894.8085	94.40878	94.45058	98.9234	97.64051
896.737	94.11909	94.67618	98.9695	97.67713
898.6655	93.64503	94.86837	99.00243	97.56082
900.5939	93.342	94.83934	99.01773	97.36467
902.5224	93.11324	94.64046	99.00108	97.25116
904.4509	92.93161	94.31557	98.91998	97.23198
906.3793	92.86922	93.84601	98.80366	97.21581
908.3078	92.59948	93.17807	98.74064	97.1287

910.2363	92.41234	92.252	98.7273	97.07653
912.1647	92.22388	91.43886	98.68641	97.01384
914.0932	92.00206	90.90436	98.57272	96.78692
916.0217	92.07456	90.52016	98.42233	96.40824
917.9501	92.18025	90.18027	98.26883	96.09728
919.8786	92.12258	89.85334	98.11112	95.96197
921.8071	92.12362	89.57542	97.94086	95.90998
923.7355	92.33522	89.33205	97.74742	95.86901
925.664	92.70236	89.27821	97.53811	95.72378
927.5925	93.08215	89.45766	97.33575	95.44814
929.5209	93.21168	89.9147	97.15528	95.14536
931.4494	93.39305	90.54852	97.0155	94.9571
933.3779	93.47503	91.20679	96.91067	94.88454
935.3063	93.39571	91.75432	96.84138	94.84646
937.2348	93.36314	92.20077	96.80662	94.89394
939.1633	93.2748	92.6604	96.78935	95.12519
941.0917	93.29169	93.26488	96.75624	95.59595
943.0202	93.48897	93.92772	96.71226	96.17126
944.9487	93.69786	94.44308	96.71228	96.65133
946.8771	93.75505	94.55869	96.7272	96.92868
948.8056	93.69132	94.49536	96.72324	97.01478
950.7341	93.71965	94.29516	96.69339	96.93825
952.6625	93.80625	93.71994	96.659	96.69516
954.591	93.59456	92.85617	96.58539	96.39703
956.5195	93.36222	92.03458	96.47995	96.13434
958.4479	93.03298	91.18468	96.36511	95.90113
960.3764	92.68694	90.31303	96.23301	95.55353
962.3049	92.51224	89.43152	96.0426	95.07377
964.2333	92.33855	88.55926	95.80158	94.61951
966.1618	92.1621	87.59691	95.54988	94.37362
968.0903	92.0389	86.63728	95.27377	94.2159
970.0187	92.09567	85.82578	94.94976	93.98461
971.9472	92.20484	85.24837	94.6083	93.78573
973.8757	92.24683	84.97411	94.2898	93.75036
975.8041	92.13754	84.73176	93.98508	93.83244
977.7326	91.85737	84.43577	93.68543	93.88447
979.6611	91.41019	84.22702	93.42876	93.87492
981.5895	91.04574	84.23622	93.22061	93.76389
983.518	90.77477	84.34733	92.99207	93.56361
985.4465	90.39085	84.32966	92.66782	93.32277
987.3749	90.05313	84.13914	92.29932	93.07681
989.3034	89.79668	83.86742	91.93056	92.89582
991.2319	89.39148	83.42757	91.56917	92.76074

993.1603	88.84358	82.72472	91.17061	92.66409
995.0888	88.28738	81.97775	90.72691	92.50003
997.0173	87.78111	81.20706	90.20839	92.25053
998.9457	87.20277	80.2663	89.59816	91.96535
1000.874	86.32571	79.15714	88.90789	91.76693
1002.803	85.3782	78.01637	88.1268	91.58137
1004.731	84.43599	76.73193	87.2098	91.24623
1006.66	83.53789	75.31252	86.1677	90.65326
1008.588	82.59548	73.72227	85.133	89.95982
1010.517	81.62238	72.07037	84.20732	89.35648
1012.445	80.65218	70.39108	83.33396	88.8218
1014.373	79.72379	68.71766	82.48375	88.24202
1016.302	78.90956	67.08821	81.71777	87.61277
1018.23	78.09072	65.52819	81.09218	87.03092
1020.159	77.34615	64.15365	80.58432	86.43524
1022.087	76.56429	62.91126	80.1469	85.77088
1024.016	75.62601	61.9002	79.76431	85.11771
1025.944	74.77605	61.20777	79.47308	84.69379
1027.873	74.13161	60.92211	79.28448	84.60095
1029.801	73.533	60.84805	79.16464	84.61648
1031.73	72.9026	60.67545	79.04893	84.56508
1033.658	72.35493	60.48798	78.93813	84.42687
1035.587	71.93064	60.50998	78.87951	84.3191
1037.515	71.55962	60.69241	78.90091	84.27936
1039.444	71.28605	60.96887	78.9518	84.19394
1041.372	71.00307	61.33775	78.95744	83.9285
1043.301	70.75657	61.67959	78.92366	83.54484
1045.229	70.39619	61.83049	78.92023	83.20328
1047.157	69.90789	61.84053	78.98708	83.04072
1049.086	69.57634	61.88306	79.07369	83.03003
1051.014	69.38062	61.85382	79.11152	83.06456
1052.943	69.02663	61.6764	79.13095	83.02239
1054.871	68.61009	61.26498	79.18846	82.9144
1056.8	68.33674	60.76723	79.29519	82.76229
1058.728	68.09255	60.0572	79.41043	82.47843
1060.657	67.8689	58.99701	79.4815	82.0911
1062.585	67.794	57.74011	79.47786	81.7707
1064.514	67.56625	56.39	79.43164	81.69309
1066.442	67.16764	54.94172	79.38951	81.80256
1068.371	66.79529	53.482	79.37451	81.87972
1070.299	66.46564	52.18545	79.37912	81.85513
1072.228	66.22322	51.17055	79.42443	81.78171
1074.156	66.09515	50.32132	79.52485	81.7522

1076.084	65.95356	49.6231	79.65419	81.76896
1078.013	65.68838	48.97298	79.77067	81.7995
1079.941	65.42905	48.49947	79.90188	81.89532
1081.87	65.24831	48.29496	80.09076	82.11653
1083.798	65.21301	48.36856	80.32925	82.47599
1085.727	65.04621	48.54312	80.55785	82.95849
1087.655	64.64137	48.86036	80.75059	83.50086
1089.584	64.39028	49.36715	80.9364	83.97193
1091.512	64.14518	50.12553	81.1415	84.28465
1093.441	63.9656	51.1635	81.35654	84.53347
1095.369	63.92478	52.50124	81.58425	84.79453
1097.298	63.80563	53.87897	81.81733	85.00348
1099.226	63.61003	55.18336	82.03111	85.15326
1101.155	63.57631	56.37773	82.22464	85.29676
1103.083	63.67815	57.52131	82.41298	85.43028
1105.011	63.83894	58.39648	82.60136	85.44418
1106.94	64.04345	59.05146	82.78658	85.29933
1108.868	64.27223	59.57626	82.96312	85.12626
1110.797	64.43539	59.90932	83.12399	85.01673
1112.725	64.62947	60.1116	83.27342	84.92684
1114.654	65.13313	60.39738	83.43973	84.82747
1116.582	65.77065	60.7456	83.64652	84.83297
1118.511	66.32563	60.95047	83.90358	84.9301
1120.439	66.77722	61.02593	84.16097	84.96729
1122.368	67.10761	61.18415	84.39687	84.96566
1124.296	67.39445	61.26571	84.61366	85.07954
1126.225	67.73952	61.1828	84.83345	85.29479
1128.153	68.08105	60.91119	85.04439	85.45891
1130.082	68.23221	60.53821	85.23083	85.57228
1132.01	68.13464	60.02272	85.39268	85.70384
1133.938	68.05567	59.40296	85.56312	85.85617
1135.867	67.99308	58.63993	85.74362	85.94474
1137.795	67.76609	57.7807	85.89169	85.96397
1139.724	67.61023	56.80398	85.96002	85.94132
1141.652	67.47228	55.7386	85.93458	85.8283
1143.581	67.14153	54.53711	85.84553	85.61059
1145.509	66.79107	53.20226	85.73899	85.41343
1147.438	66.47317	51.80939	85.62201	85.39818
1149.366	66.16522	50.51329	85.46242	85.49109
1151.295	65.75361	49.22768	85.26002	85.53429
1153.223	65.21971	48.06075	85.05512	85.53624
1155.152	64.79338	47.09965	84.87177	85.56043
1157.08	64.51608	46.31889	84.71075	85.55264

1159.009	64.24951	45.7615	84.56911	85.51209
1160.937	64.09816	45.3511	84.4529	85.53496
1162.865	63.93176	44.99608	84.36389	85.62987
1164.794	63.78518	44.72792	84.28283	85.6669
1166.722	63.62344	44.60406	84.17578	85.56525
1168.651	63.52275	44.66393	84.06697	85.335
1170.579	63.46078	44.74731	84.00183	85.02717
1172.508	63.33767	44.73095	83.98715	84.70797
1174.436	63.18941	44.64367	83.98798	84.42968
1176.365	63.03296	44.4662	83.97712	84.2163
1178.293	62.87725	44.10104	83.93777	84.06623
1180.222	62.60968	43.60959	83.8823	83.92472
1182.15	62.22305	42.99158	83.83722	83.74314
1184.079	61.93383	42.26044	83.80988	83.55509
1186.007	61.69571	41.48066	83.77753	83.44531
1187.936	61.41179	40.70744	83.72363	83.45184
1189.864	61.03283	39.9352	83.6584	83.49499
1191.792	60.68321	39.08664	83.61121	83.4886
1193.721	60.27637	38.30643	83.58825	83.4072
1195.649	59.67402	37.70287	83.56255	83.29298
1197.578	58.93509	37.13538	83.50626	83.16457
1199.506	58.2604	36.63704	83.41288	83.00296
1201.435	57.7619	36.18122	83.31433	82.79997
1203.363	57.34135	35.7187	83.21264	82.52438
1205.292	56.85898	35.2773	83.09553	82.16077
1207.22	56.34998	34.81942	82.95081	81.82486
1209.149	55.80997	34.32857	82.7722	81.59832
1211.077	55.3258	33.83777	82.5554	81.40933
1213.006	54.79979	33.27291	82.30785	81.15832
1214.934	54.25463	32.62949	82.03676	80.91325
1216.863	53.68843	31.91561	81.73737	80.76791
1218.791	53.09206	31.13021	81.42209	80.67926
1220.719	52.5574	30.26026	81.10565	80.56878
1222.648	52.04877	29.32637	80.79634	80.45747
1224.576	51.41637	28.30301	80.50174	80.4137
1226.505	50.74615	27.29584	80.24947	80.41
1228.433	50.10682	26.33661	80.07101	80.33111
1230.362	49.6342	25.5078	79.98137	80.13124
1232.29	49.27909	24.85175	79.95193	79.85002
1234.219	49.02864	24.30211	79.95731	79.56556
1236.147	48.86905	23.77566	80.00425	79.31874
1238.076	48.7021	23.28808	80.10962	79.12608
1240.004	48.43679	22.94248	80.25774	78.99608

1241.933	48.13846	22.7833	80.40279	78.9002
1243.861	48.0006	22.74747	80.48037	78.80954
1245.79	47.94159	22.82008	80.45124	78.74967
1247.718	47.91098	22.9974	80.33113	78.67025
1249.646	47.96536	23.28243	80.18719	78.54948
1251.575	48.12131	23.63654	80.05205	78.43658
1253.503	48.36588	24.00235	79.92997	78.42343
1255.432	48.72028	24.36318	79.83572	78.46246
1257.36	49.20002	24.70458	79.8026	78.51187
1259.289	49.67438	25.04127	79.84093	78.57845
1261.217	50.14501	25.36909	79.94084	78.67471
1263.146	50.63007	25.73221	80.09709	78.77845
1265.074	51.14125	26.07317	80.33005	78.88298
1267.003	51.70897	26.36462	80.65388	78.97972
1268.931	52.24004	26.66078	81.04196	79.07145
1270.86	52.61538	26.87701	81.44497	79.18303
1272.788	52.95514	27.10244	81.84835	79.32249
1274.717	53.34819	27.38842	82.2644	79.45391
1276.645	53.85924	27.73425	82.67522	79.57391
1278.573	54.35175	28.04649	83.03684	79.71038
1280.502	54.69696	28.29042	83.31484	79.83242
1282.43	54.88483	28.46315	83.49857	79.96585
1284.359	54.95613	28.57985	83.62092	80.19045
1286.287	55.00608	28.67925	83.72716	80.47388
1288.216	55.10034	28.88673	83.83764	80.71192
1290.144	55.23421	29.10365	83.93554	80.87609
1292.073	55.30491	29.23856	84.01241	80.98263
1294.001	55.33995	29.37047	84.07944	81.01026
1295.93	55.45019	29.39649	84.15642	81.01314
1297.858	55.5426	29.22326	84.25713	81.03055
1299.787	55.56862	28.96721	84.37363	81.0561
1301.715	55.60542	28.71657	84.4699	81.06435
1303.644	55.55615	28.3731	84.52998	81.05226
1305.572	55.46966	27.98643	84.5751	81.01736
1307.5	55.47346	27.58827	84.6183	81.00487
1309.429	55.4886	27.15525	84.64537	81.03531
1311.357	55.52481	26.65068	84.68369	81.05265
1313.286	55.60213	26.10408	84.77518	81.03817
1315.214	55.64382	25.62798	84.88963	81.05827
1317.143	55.68668	25.20031	84.99178	81.16607
1319.071	55.79828	24.83929	85.10189	81.38275
1321	55.93803	24.57182	85.23134	81.69831
1322.928	56.07092	24.35162	85.33549	82.01353

1324.857	56.09972	24.08349	85.39111	82.19474
1326.785	56.10707	23.8396	85.42196	82.24928
1328.714	56.17335	23.699	85.45488	82.26174
1330.642	56.31986	23.64946	85.48088	82.25414
1332.571	56.50143	23.68335	85.48089	82.21022
1334.499	56.73236	23.87213	85.46875	82.15029
1336.427	56.87778	24.15175	85.46756	82.05179
1338.356	56.9566	24.50608	85.4531	81.89848
1340.284	56.93894	24.92173	85.38177	81.71529
1342.213	56.81387	25.3723	85.29297	81.54712
1344.141	56.70144	25.88711	85.25277	81.47921
1346.07	56.58825	26.44477	85.25109	81.53276
1347.998	56.47646	26.92402	85.2291	81.61968
1349.927	56.34692	27.30317	85.18247	81.66524
1351.855	56.10541	27.65333	85.11145	81.71482
1353.784	55.83868	27.93557	85.00746	81.76648
1355.712	55.57457	28.04124	84.89453	81.78721
1357.641	55.27266	27.99097	84.78725	81.80302
1359.569	54.93858	27.81729	84.64761	81.78207
1361.498	54.57059	27.51667	84.46066	81.63924
1363.426	54.16006	27.0499	84.2658	81.38433
1365.354	53.65075	26.45218	84.07749	81.11581
1367.283	53.04449	25.76567	83.87702	80.86295
1369.211	52.39793	24.93828	83.63487	80.58204
1371.14	51.63079	23.9193	83.3549	80.22967
1373.068	50.77118	22.88777	83.06579	79.78101
1374.997	49.90695	21.90503	82.79766	79.27259
1376.925	49.21039	20.93737	82.54603	78.79933
1378.854	48.58862	19.98944	82.31793	78.41524
1380.782	47.91702	19.11569	82.14616	78.11282
1382.711	47.25556	18.33574	82.02396	77.85974
1384.639	46.65878	17.60486	81.90145	77.61049
1386.568	46.09915	16.93397	81.75079	77.36573
1388.496	45.65219	16.41321	81.60696	77.16635
1390.424	45.41963	15.95579	81.511	77.02602
1392.353	45.27385	15.60651	81.47365	76.90089
1394.281	45.21658	15.46544	81.47931	76.81095
1396.21	45.23901	15.51066	81.51241	76.75735
1398.138	45.24381	15.59029	81.57518	76.74026
1400.067	45.41972	15.71066	81.65538	76.78749
1401.995	45.665	15.94733	81.72397	76.94017
1403.924	45.89407	16.24376	81.77244	77.17126
1405.852	46.21015	16.52793	81.8228	77.43347

1407.781	46.6393	16.80285	81.89476	77.69657
1409.709	47.06815	17.10901	81.99997	77.93833
1411.638	47.47028	17.38504	82.13402	78.15493
1413.566	47.83921	17.56883	82.27541	78.35562
1415.495	48.12768	17.56683	82.39563	78.54705
1417.423	48.2249	17.43045	82.4962	78.73747
1419.351	48.22482	17.33606	82.57223	78.92911
1421.28	48.2079	17.22292	82.61336	79.08408
1423.208	48.19277	17.02002	82.58973	79.19799
1425.137	48.03784	16.67659	82.49073	79.27043
1427.065	47.81926	16.22805	82.32412	79.23133
1428.994	47.53841	15.72418	82.09797	79.02385
1430.922	47.16254	15.12941	81.83357	78.67495
1432.851	46.75792	14.56223	81.57489	78.24404
1434.779	46.24875	14.10583	81.35631	77.76876
1436.708	45.55143	13.62333	81.15054	77.31596
1438.636	44.85481	13.19389	80.92118	76.93826
1440.565	44.14158	12.7925	80.67888	76.61352
1442.493	43.43855	12.41887	80.46662	76.29994
1444.422	42.89711	12.19053	80.30627	75.99749
1446.35	42.47169	12.14011	80.21609	75.73114
1448.278	42.01022	12.20899	80.19566	75.48402
1450.207	41.73542	12.265	80.22053	75.25401
1452.135	41.67091	12.36272	80.25486	75.1055
1454.064	41.71266	12.62337	80.30838	75.11007
1455.992	41.90879	13.02242	80.41062	75.25941
1457.921	42.24522	13.51159	80.56846	75.4946
1459.849	42.61638	14.10518	80.77378	75.74517
1461.778	42.96996	14.78344	80.99665	76.00156
1463.706	43.33809	15.6178	81.20222	76.29194
1465.635	43.81633	16.58177	81.35552	76.58184
1467.563	44.40659	17.53794	81.45931	76.81117
1469.492	44.96946	18.37706	81.55275	77.04023
1471.42	45.35137	19.12109	81.66066	77.37437
1473.349	45.59929	19.82464	81.75436	77.7765
1475.277	45.65377	20.42488	81.77789	78.08582
1477.205	45.49124	20.81139	81.70018	78.27994
1479.134	45.13968	20.98515	81.51268	78.3839
1481.062	44.45093	20.9468	81.20038	78.39511
1482.991	43.45363	20.66609	80.76069	78.27949
1484.919	42.46383	20.30255	80.19546	78.02848
1486.848	41.34957	19.76349	79.5525	77.62592
1488.776	40.10625	19.11604	78.89197	77.13389

1490.705	38.80734	18.35007	78.26724	76.65195
1492.633	37.39647	17.30177	77.69091	76.13732
1494.562	35.99601	16.10229	77.15893	75.49224
1496.49	34.80635	14.93042	76.67261	74.74489
1498.419	33.60313	13.78533	76.24198	74.03814
1500.347	32.33078	12.71106	75.88098	73.43975
1502.276	31.22798	11.65507	75.58715	72.96565
1504.204	30.20066	10.66429	75.36306	72.55963
1506.132	29.26013	9.84732	75.22324	72.21064
1508.061	28.51719	9.15802	75.16782	71.94099
1509.989	27.96717	8.54427	75.17697	71.73891
1511.918	27.49902	8.0354	75.22741	71.50399
1513.846	27.10116	7.57383	75.30383	71.21966
1515.775	26.65845	7.08104	75.36878	70.97376
1517.703	26.15964	6.74326	75.39426	70.79291
1519.632	25.93221	6.51049	75.372	70.6354
1521.56	25.85771	6.21589	75.29644	70.41635
1523.489	25.79671	5.84213	75.18742	70.15531
1525.417	25.76261	5.52685	75.12048	69.90521
1527.346	25.70298	5.2991	75.12168	69.69306
1529.274	25.69947	5.23574	75.13351	69.44945
1531.203	25.83883	5.27027	75.09669	69.1253
1533.131	25.88572	5.22086	75.03184	68.77888
1535.059	25.83305	5.10624	74.96742	68.51422
1536.988	25.81252	5.03974	74.89235	68.37286
1538.916	25.98389	5.03295	74.8612	68.3318
1540.845	26.29891	5.17164	74.9055	68.39029
1542.773	26.57389	5.36996	74.99576	68.51593
1544.702	26.72167	5.4277	75.05824	68.65926
1546.63	26.9771	5.44633	75.11648	68.79324
1548.559	27.32128	5.50239	75.22822	68.9585
1550.487	27.61505	5.62229	75.42797	69.18297
1552.416	27.96978	5.8272	75.68721	69.4838
1554.344	28.39171	5.95003	75.9482	69.84437
1556.273	28.84684	6.02869	76.21287	70.23559
1558.201	29.31928	6.19764	76.49494	70.62204
1560.13	29.84298	6.37284	76.77542	70.94657
1562.058	30.42476	6.50922	77.0538	71.27239
1563.986	31.121	6.76349	77.3479	71.65313
1565.915	31.93018	7.0195	77.64423	72.03564
1567.843	32.77169	7.16981	77.92971	72.33896
1569.772	33.6973	7.30956	78.21	72.62226
1571.7	34.64692	7.54575	78.46518	72.95954

1573.629	35.53331	7.80924	78.66338	73.32361
1575.557	36.30368	8.12995	78.82269	73.65182
1577.486	36.97858	8.42749	79.01284	73.93139
1579.414	37.56379	8.68035	79.20229	74.11125
1581.343	38.06901	8.94434	79.311	74.17596
1583.271	38.51941	9.29318	79.32419	74.18645
1585.2	38.93547	9.55302	79.32478	74.17913
1587.128	39.18797	9.71388	79.3292	74.10825
1589.057	39.19163	9.78355	79.28413	73.9217
1590.985	38.98952	9.768	79.13717	73.62277
1592.913	38.69103	9.69082	78.88123	73.26011
1594.842	38.17585	9.57159	78.56692	72.87031
1596.77	37.51671	9.42997	78.20861	72.45829
1598.699	36.77235	9.22501	77.78397	72.02002
1600.627	35.87859	8.85453	77.2824	71.62499
1602.556	34.86588	8.43634	76.75856	71.2598
1604.484	33.77641	8.0097	76.25135	70.8717
1606.413	32.62579	7.53233	75.76771	70.46617
1608.341	31.42911	7.06743	75.29181	70.10222
1610.27	30.27161	6.64276	74.82155	69.78642
1612.198	29.13931	6.16204	74.37682	69.46601
1614.127	28.08438	5.56166	73.9715	69.124
1616.055	27.11658	5.01628	73.61017	68.76533
1617.984	26.1314	4.57902	73.2924	68.37651
1619.912	25.06263	4.16592	73.00699	67.96439
1621.84	24.08332	3.72209	72.74714	67.59469
1623.769	23.39768	3.36427	72.52691	67.26743
1625.697	22.80655	3.1836	72.35671	66.90492
1627.626	22.167	3.04258	72.2023	66.47435
1629.554	21.5057	2.86349	72.05631	66.0146
1631.483	21.05215	2.67474	71.93828	65.58963
1633.411	20.73628	2.52439	71.88077	65.23674
1635.34	20.46306	2.40828	71.85054	64.95932
1637.268	20.25396	2.33207	71.81781	64.70752
1639.197	20.13574	2.27996	71.78629	64.44942
1641.125	19.97953	2.24775	71.79006	64.19459
1643.054	19.77715	2.20088	71.82832	64.0054
1644.982	19.60613	2.10676	71.87747	63.89015
1646.911	19.45816	2.01334	71.90744	63.81847
1648.839	19.38095	2.03475	71.89015	63.8
1650.767	19.34369	2.12256	71.85837	63.82049
1652.696	19.36198	2.24483	71.87014	63.85401
1654.624	19.35497	2.37635	71.94295	63.88496

1656.553	19.34395	2.38666	72.02386	63.91114
1658.481	19.29684	2.32018	72.07367	63.94226
1660.41	19.21399	2.25582	72.10306	64.04855
1662.338	19.26941	2.32489	72.16674	64.26102
1664.267	19.29187	2.44118	72.28476	64.5332
1666.195	19.30158	2.51624	72.40594	64.82191
1668.124	19.45344	2.60416	72.50515	65.09335
1670.052	19.6722	2.80337	72.59721	65.35909
1671.981	19.792	2.99527	72.70478	65.66968
1673.909	19.84365	2.95045	72.80011	66.04534
1675.838	19.93854	2.79897	72.88111	66.39488
1677.766	20.24428	2.72253	72.95419	66.66323
1679.694	20.57067	2.79307	73.04284	66.91372
1681.623	20.77069	2.94144	73.12439	67.21124
1683.551	21.03624	3.12169	73.15708	67.54404
1685.48	21.44678	3.25534	73.1205	67.87601
1687.408	21.83466	3.38315	73.08484	68.24375
1689.337	22.21574	3.56095	73.1191	68.63899
1691.265	22.7296	3.78516	73.21532	69.02904
1693.194	23.38992	4.09842	73.29335	69.40491
1695.122	24.11476	4.51129	73.33983	69.79026
1697.051	24.78672	4.91703	73.42827	70.23151
1698.979	25.46057	5.35616	73.57099	70.72803
1700.908	26.16104	5.82883	73.71641	71.29332
1702.836	26.98857	6.27656	73.83237	71.89303
1704.765	27.92688	6.78443	73.93462	72.47124
1706.693	28.9993	7.36198	74.04273	72.99356
1708.621	30.26584	7.98472	74.18636	73.51779
1710.55	31.42501	8.65035	74.39068	74.09856
1712.478	32.5567	9.28925	74.66143	74.72496
1714.407	33.85752	9.90281	74.98023	75.26822
1716.335	35.12677	10.45079	75.31142	75.67179
1718.264	36.34806	10.9914	75.64822	76.02625
1720.192	37.49839	11.54563	75.99731	76.43307
1722.121	38.66583	12.23033	76.3685	76.85702
1724.049	39.89154	13.04093	76.77375	77.25142
1725.978	41.13319	13.87289	77.24191	77.6702
1727.906	42.3983	14.62749	77.75664	78.13464
1729.835	43.77073	15.36363	78.29007	78.63647
1731.763	45.06649	16.07635	78.81841	79.15026
1733.692	46.16317	16.80682	79.35666	79.67488
1735.62	47.18406	17.5334	79.89758	80.23593
1737.548	48.30736	18.25775	80.39312	80.7875

1739.477	49.49174	19.05167	80.83492	81.31692
1741.405	50.54028	19.84924	81.26476	81.81835
1743.334	51.46246	20.63836	81.70815	82.34671
1745.262	52.35003	21.48263	82.15503	82.89743
1747.191	53.23206	22.29462	82.61431	83.47343
1749.119	54.07883	23.03619	83.07757	84.04049
1751.048	54.81373	23.74018	83.53213	84.59583
1752.976	55.55935	24.41923	83.96397	85.08351
1754.905	56.33148	25.11178	84.37341	85.4577
1756.833	56.99374	25.82811	84.77132	85.73195
1758.762	57.73159	26.45351	85.16195	85.9698
1760.69	58.55288	27.08937	85.53148	86.19457
1762.619	59.24419	27.77493	85.88937	86.419
1764.547	59.92521	28.51636	86.2687	86.68637
1766.475	60.63827	29.24836	86.66777	87.00929
1768.404	61.29442	29.96254	87.04292	87.37683
1770.332	61.93707	30.63469	87.37063	87.75559
1772.261	62.61058	31.29147	87.67467	88.13784
1774.189	63.26059	31.98648	87.97599	88.4829
1776.118	63.83018	32.66219	88.26851	88.73787
1778.046	64.37331	33.28473	88.54859	88.93182
1779.975	65.00819	33.88812	88.83298	89.16963
1781.903	65.73268	34.53935	89.13473	89.47946
1783.832	66.55446	35.24783	89.44968	89.76203
1785.76	67.33739	35.92353	89.74597	89.98034
1787.689	67.98166	36.55336	90.00841	90.17332
1789.617	68.47263	37.23596	90.25694	90.39641
1791.546	68.92294	37.95408	90.52017	90.60943
1793.474	69.41946	38.57343	90.78835	90.77071
1795.402	69.91051	39.12936	91.02606	90.86616
1797.331	70.35828	39.6845	91.22819	90.94927
1799.259	70.84981	40.25255	91.43258	91.06468
1801.188	71.35152	40.86004	91.65973	91.19973
1803.116	71.87114	41.42932	91.88933	91.33406
1805.045	72.44804	41.97923	92.08826	91.47473
1806.973	72.93504	42.59019	92.24223	91.66144
1808.902	73.38004	43.19153	92.36874	91.93155
1810.83	73.87259	43.80118	92.519	92.2319
1812.759	74.39057	44.45804	92.69823	92.42294
1814.687	74.86149	45.13965	92.87667	92.49007
1816.616	75.3868	45.79271	93.04813	92.5641
1818.544	75.90826	46.39888	93.23405	92.71198
1820.473	76.35032	46.99055	93.4263	92.83704

1822.401	76.79211	47.58498	93.59916	92.9005
1824.329	77.24253	48.17902	93.75021	92.94242
1826.258	77.68237	48.78669	93.89527	92.98372
1828.186	78.14381	49.3941	94.03278	93.02696
1830.115	78.60713	49.98011	94.16744	93.08677
1832.043	79.00612	50.51068	94.3166	93.15651
1833.972	79.36012	51.07113	94.47468	93.19341
1835.9	79.67088	51.67789	94.62818	93.22122
1837.829	80.02204	52.2608	94.78247	93.30681
1839.757	80.36855	52.80768	94.92376	93.4694
1841.686	80.65086	53.36097	95.03469	93.67728
1843.614	80.93452	53.90241	95.12735	93.91438
1845.543	81.25678	54.43164	95.21674	94.18014
1847.471	81.58132	54.95785	95.31298	94.43976
1849.4	81.94135	55.45831	95.43807	94.63661
1851.328	82.27901	55.95911	95.58292	94.78541
1853.256	82.51266	56.42937	95.717	94.93779
1855.185	82.78091	56.87154	95.84161	95.11841
1857.113	83.09637	57.34508	95.97285	95.27803
1859.042	83.40042	57.84154	96.10681	95.39618
1860.97	83.72034	58.327	96.23946	95.49261
1862.899	84.0053	58.74624	96.37327	95.5484
1864.827	84.24258	59.13738	96.49396	95.56934
1866.756	84.42799	59.52386	96.59718	95.62759
1868.684	84.59519	59.92905	96.70579	95.75199
1870.613	84.75486	60.31218	96.83802	95.87521
1872.541	84.89827	60.63446	96.97391	95.97002
1874.47	85.10117	60.92161	97.09087	96.08395
1876.398	85.32879	61.27991	97.18116	96.24667
1878.327	85.56633	61.68865	97.23709	96.43458
1880.255	85.80533	62.10678	97.26583	96.59222
1882.183	85.97884	62.57183	97.28879	96.7122
1884.112	86.20522	63.0914	97.30323	96.80459
1886.04	86.47636	63.59293	97.30879	96.83768
1887.969	86.7588	64.04861	97.32845	96.82793
1889.897	87.01759	64.54714	97.36998	96.81637
1891.826	87.26797	65.08356	97.40625	96.76827
1893.754	87.46452	65.58017	97.43271	96.6418
1895.683	87.54516	66.03336	97.45626	96.45193
1897.611	87.51457	66.46266	97.48639	96.28511
1899.54	87.55416	66.84675	97.53605	96.15191
1901.468	87.6776	67.20789	97.6133	96.03249
1903.397	87.81021	67.63759	97.70206	95.93059

1905.325	87.96922	68.05276	97.80408	95.86995
1907.254	88.20688	68.39746	97.91797	95.83228
1909.182	88.44196	68.70483	98.02516	95.80187
1911.11	88.66365	68.99631	98.0996	95.81497
1913.039	88.80899	69.27344	98.14189	95.89829
1914.967	88.89277	69.54996	98.1637	96.04467
1916.896	88.94852	69.82133	98.1835	96.23365
1918.824	88.94365	70.05137	98.22143	96.4344
1920.753	88.9568	70.28625	98.281	96.64379
1922.681	88.98787	70.54263	98.3481	96.84017
1924.61	89.11251	70.77229	98.4191	96.97981
1926.538	89.35188	71.03387	98.49403	97.07991
1928.467	89.50869	71.39097	98.55567	97.19716
1930.395	89.63555	71.7207	98.59211	97.33946
1932.324	89.71099	71.97842	98.62043	97.46247
1934.252	89.81778	72.28368	98.6462	97.54609
1936.181	89.91199	72.66911	98.66329	97.61343
1938.109	90.05819	73.02193	98.68793	97.62488
1940.037	90.17306	73.34209	98.73505	97.5757
1941.966	90.22848	73.6516	98.78625	97.48563
1943.894	90.32146	73.9558	98.82473	97.38978
1945.823	90.53185	74.24359	98.84315	97.29424
1947.751	90.80434	74.46619	98.84669	97.2402
1949.68	91.10491	74.71797	98.83777	97.25181
1951.608	91.36597	74.96514	98.82894	97.32068
1953.537	91.58436	75.22642	98.82326	97.41805
1955.465	91.7483	75.46262	98.83231	97.505
1957.394	91.85223	75.66304	98.86979	97.56167
1959.322	91.88005	75.90643	98.92219	97.61068
1961.251	91.84611	76.15198	98.96756	97.67202
1963.179	91.72974	76.365	98.996	97.72486
1965.108	91.57515	76.5358	99.01523	97.74665
1967.036	91.5145	76.70631	99.02327	97.79583
1968.964	91.49952	76.90684	99.02072	97.88167
1970.893	91.51766	77.12997	99.01522	97.93527
1972.821	91.52822	77.40549	99.00695	97.9223
1974.75	91.40424	77.65578	99.00402	97.89536
1976.678	91.28996	77.86065	99.0223	97.85419
1978.607	91.27094	78.11293	99.06242	97.76497
1980.535	91.3094	78.41478	99.10474	97.62974
1982.464	91.3159	78.67249	99.14411	97.51219
1984.392	91.35376	78.88594	99.19235	97.46686
1986.321	91.40546	79.07887	99.24988	97.54213

1988.249	91.44824	79.24989	99.29803	97.68935
1990.178	91.50147	79.41328	99.32015	97.82988
1992.106	91.65348	79.50499	99.31799	97.92712
1994.035	91.90837	79.55003	99.30526	97.98614
1995.963	92.11702	79.67657	99.28565	97.98827
1997.891	92.2344	79.80992	99.24433	97.92108
1999.82	92.34788	79.92047	99.18815	97.82305
2001.748	92.48161	79.97787	99.13863	97.74932
2003.677	92.64439	80.03241	99.10526	97.71648
2005.605	92.77501	80.13025	99.0689	97.72106
2007.534	92.85146	80.24105	99.04077	97.69828
2009.462	92.86678	80.37701	99.04199	97.62753
2011.391	92.94722	80.52679	99.06267	97.56734
2013.319	93.07508	80.67062	99.08208	97.55695
2015.248	93.12446	80.81255	99.10129	97.52621
2017.176	93.13139	80.99096	99.13206	97.43289
2019.105	93.25527	81.19297	99.1626	97.35177
2021.033	93.34842	81.35101	99.19013	97.33504
2022.962	93.3694	81.51589	99.22008	97.36302
2024.89	93.33547	81.65923	99.26001	97.41871
2026.818	93.2038	81.74747	99.29484	97.47107
2028.747	93.04675	81.78443	99.29501	97.47775
2030.675	92.97404	81.84331	99.25316	97.43968
2032.604	92.9687	81.91958	99.19588	97.37233
2034.532	92.91574	82.02181	99.14755	97.2865
2036.461	92.81056	82.13347	99.10809	97.21463
2038.389	92.76981	82.21439	99.0779	97.21769
2040.318	92.74929	82.30463	99.06711	97.31304
2042.246	92.65821	82.37911	99.07575	97.41384
2044.175	92.49975	82.41999	99.0921	97.43117
2046.103	92.38033	82.50726	99.11366	97.3852
2048.032	92.30441	82.59725	99.12673	97.3675
2049.96	92.2082	82.69574	99.11734	97.4075
2051.889	92.05899	82.84698	99.0951	97.45321
2053.817	91.90792	83.01333	99.07394	97.45943
2055.746	91.83747	83.11951	99.04896	97.44946
2057.674	91.82765	83.15657	99.01384	97.4231
2059.603	91.75936	83.14867	98.99167	97.32293
2061.531	91.71409	83.16734	98.99341	97.13971
2063.459	91.69197	83.16264	98.9962	96.96569
2065.388	91.68072	83.06197	98.9709	96.85042
2067.316	91.60671	82.95795	98.92087	96.78286
2069.245	91.48798	82.87831	98.8863	96.78461

2071.173	91.43604	82.78571	98.8933	96.8267
2073.102	91.42539	82.77308	98.9278	96.86818
2075.03	91.50352	82.80396	98.96232	96.92407
2076.959	91.66238	82.86206	98.98443	97.04466
2078.887	91.77243	82.96649	99.00486	97.20665
2080.816	91.83755	83.09004	99.01932	97.31836
2082.744	91.90304	83.17811	99.01502	97.32985
2084.673	92.02621	83.28159	98.98248	97.25979
2086.601	92.17312	83.40533	98.94323	97.17689
2088.53	92.29044	83.50604	98.91588	97.12193
2090.458	92.36987	83.60432	98.89263	97.06986
2092.386	92.4911	83.72073	98.85686	97.00757
2094.315	92.53941	83.86815	98.81287	96.96204
2096.243	92.52767	84.05685	98.78065	96.91404
2098.172	92.56702	84.16879	98.77177	96.82455
2100.1	92.72182	84.22346	98.77247	96.72189
2102.029	92.923	84.24789	98.75024	96.66972
2103.957	93.063	84.29211	98.70347	96.69019
2105.886	93.10963	84.35127	98.67235	96.7701
2107.814	93.11832	84.40643	98.67407	96.89763
2109.743	93.04078	84.42063	98.68821	97.01101
2111.671	92.90828	84.43254	98.68769	97.11099
2113.6	92.80888	84.45383	98.6724	97.23184
2115.528	92.7087	84.47305	98.64977	97.37931
2117.457	92.67436	84.55123	98.63107	97.50802
2119.385	92.58846	84.65314	98.61487	97.57284
2121.313	92.43194	84.6918	98.60338	97.56273
2123.242	92.28949	84.69035	98.61563	97.4975
2125.17	92.19887	84.73457	98.662	97.45644
2127.099	92.12656	84.79982	98.7105	97.46269
2129.027	92.00124	84.85212	98.73698	97.47595
2130.956	91.88507	84.95979	98.75594	97.47607
2132.884	91.82695	85.08572	98.79027	97.48133
2134.813	91.75706	85.17859	98.84329	97.45007
2136.741	91.67022	85.26991	98.90541	97.35712
2138.67	91.64417	85.32347	98.96492	97.25865
2140.598	91.6587	85.3736	98.99771	97.1952
2142.527	91.77331	85.4544	99.00366	97.16983
2144.455	91.96111	85.49844	99.00851	97.18356
2146.384	92.17411	85.58778	99.01697	97.25156
2148.312	92.29676	85.71362	99.0059	97.30409
2150.24	92.34396	85.85752	98.95894	97.31121
2152.169	92.38206	85.98318	98.90852	97.29437

2154.097	92.42146	86.08271	98.87488	97.29075
2156.026	92.46946	86.15039	98.8563	97.27881
2157.954	92.57344	86.21499	98.85304	97.25087
2159.883	92.69215	86.29754	98.86142	97.2356
2161.811	92.71305	86.41242	98.87804	97.24593
2163.74	92.73998	86.50401	98.88942	97.28795
2165.668	92.74461	86.53684	98.89727	97.3502
2167.597	92.68731	86.59988	98.90403	97.38953
2169.525	92.65843	86.69967	98.90097	97.37204
2171.454	92.62496	86.8387	98.88579	97.32794
2173.382	92.55116	86.93745	98.8717	97.305
2175.311	92.4127	86.99563	98.88166	97.33894
2177.239	92.2882	87.12254	98.91576	97.39121
2179.167	92.23765	87.31075	98.94631	97.40906
2181.096	92.21013	87.47459	98.96552	97.39296
2183.024	92.19731	87.5833	98.97061	97.39423
2184.953	92.17377	87.63401	98.95731	97.39585
2186.881	92.14288	87.72184	98.92235	97.35726
2188.81	92.10999	87.86362	98.90416	97.29023
2190.738	92.08066	87.97334	98.94056	97.21393
2192.667	92.11104	88.11417	99.00715	97.13266
2194.595	92.17449	88.26599	99.06668	97.04319
2196.524	92.33705	88.37925	99.1068	96.99237
2198.452	92.60427	88.44061	99.12849	97.01172
2200.381	92.85017	88.52519	99.14423	97.06336
2202.309	93.14556	88.60658	99.16049	97.12413
2204.238	93.40024	88.7471	99.16242	97.21636
2206.166	93.49998	88.90828	99.11995	97.32181
2208.094	93.62125	88.96675	99.03458	97.41043
2210.023	93.83849	88.97769	98.94936	97.49319
2211.951	94.01989	89.0483	98.90004	97.5632
2213.88	94.08887	89.14888	98.88638	97.55803
2215.808	94.07484	89.21095	98.87529	97.4846
2217.737	94.04072	89.26554	98.85196	97.42877
2219.665	94.03728	89.30353	98.83666	97.4245
2221.594	93.97019	89.38742	98.83394	97.42904
2223.522	93.91447	89.56837	98.83726	97.40025
2225.451	93.88833	89.74722	98.85593	97.37149
2227.379	93.87344	89.82617	98.89973	97.37082
2229.308	93.74033	89.86811	98.9347	97.4167
2231.236	93.56339	89.94418	98.93143	97.48867
2233.165	93.45009	90.09474	98.91388	97.52061
2235.093	93.35106	90.25515	98.90813	97.51389

2237.021	93.28967	90.38043	98.9105	97.51804
2238.95	93.27745	90.5291	98.91766	97.53277
2240.878	93.22453	90.64464	98.91716	97.52668
2242.807	93.23356	90.70232	98.89765	97.50785
2244.735	93.29195	90.75557	98.86952	97.47399
2246.664	93.36314	90.90594	98.85711	97.41958
2248.592	93.46856	91.08923	98.85806	97.36768
2250.521	93.52324	91.33069	98.8513	97.27858
2252.449	93.54263	91.51903	98.83909	97.11865
2254.378	93.65483	91.58623	98.83523	97.01055
2256.306	93.71886	91.66694	98.85895	97.04548
2258.235	93.69582	91.81225	98.90858	97.15132
2260.163	93.6184	91.96355	98.95109	97.19327
2262.092	93.57999	92.14426	98.9527	97.18372
2264.02	93.67181	92.32987	98.92292	97.20466
2265.948	93.80037	92.4247	98.88419	97.30238
2267.877	93.83835	92.46275	98.84757	97.42809
2269.805	93.93504	92.53687	98.82717	97.49973
2271.734	94.00484	92.58186	98.83108	97.5216
2273.662	94.00137	92.59629	98.83776	97.55866
2275.591	93.93702	92.65066	98.84308	97.60025
2277.519	93.9842	92.79807	98.84926	97.6148
2279.448	94.09198	92.96795	98.83709	97.60359
2281.376	94.12374	93.10728	98.79508	97.61613
2283.305	94.03427	93.20076	98.74255	97.67489
2285.233	94.06917	93.25523	98.6962	97.74325
2287.162	94.16506	93.34872	98.66766	97.73277
2289.09	94.20656	93.50015	98.65984	97.59756
2291.019	94.2045	93.71795	98.66934	97.46076
2292.947	94.25033	94.01698	98.67482	97.43327
2294.875	94.35451	94.27084	98.67184	97.44402
2296.804	94.48954	94.44223	98.66009	97.39115
2298.732	94.44907	94.50379	98.65468	97.30805
2300.661	94.40403	94.53676	98.66367	97.27486
2302.589	94.34214	94.56547	98.69991	97.24197
2304.518	94.23251	94.56483	98.77651	97.1708
2306.446	94.04916	94.591	98.87931	97.07037
2308.375	93.81183	94.68652	98.98315	96.95278
2310.303	93.55882	94.66423	99.08348	96.85596
2312.232	93.44009	94.60175	99.18191	96.81895
2314.16	93.51267	94.60088	99.23865	96.82942
2316.089	93.8308	94.72772	99.22025	96.84547
2318.017	93.94632	94.87424	99.18137	96.78281

2319.946	93.9638	95.09038	99.15538	96.6683
2321.874	94.27847	95.20485	99.12065	96.55852
2323.802	94.66949	95.36163	99.06237	96.4857
2325.731	94.93557	95.50433	99.00377	96.40236
2327.659	95.10726	95.62449	98.9301	96.34329
2329.588	95.31728	95.70922	98.79841	96.35107
2331.516	95.57477	95.76333	98.64042	96.38365
2333.445	95.69118	95.94788	98.48006	96.41923
2335.373	95.6449	96.17081	98.29332	96.48746
2337.302	95.64225	96.29102	98.12	96.59488
2339.23	95.59795	96.29259	98.01279	96.67066
2341.159	95.43472	96.41554	97.99799	96.6639
2343.087	95.29701	96.47655	98.01329	96.60399
2345.016	94.94846	96.4026	98.05781	96.54507
2346.944	94.60268	96.22041	98.07775	96.51597
2348.873	94.13371	95.9856	98.05962	96.59343
2350.801	93.61124	95.78326	98.00479	96.76191
2352.729	93.15553	95.70651	97.96738	96.91591
2354.658	92.77804	95.67082	97.98387	96.93138
2356.586	92.68214	95.53056	98.06173	96.83042
2358.515	92.92402	95.42568	98.14203	96.81459
2360.443	93.28162	95.36041	98.20695	96.96056
2362.372	93.58823	95.39605	98.2823	97.15292
2364.3	93.61193	95.45502	98.37352	97.25992
2366.229	93.73599	95.47721	98.46702	97.33589
2368.157	94.04858	95.37193	98.56041	97.42267
2370.086	94.22569	95.31615	98.65945	97.49859
2372.014	94.39402	95.25849	98.71304	97.54617
2373.942	94.66099	95.16397	98.70811	97.55745
2375.871	94.86848	95.06538	98.71737	97.53153
2377.799	94.90665	95.00732	98.76096	97.47292
2379.728	94.94128	95.00809	98.80276	97.3865
2381.656	94.87111	94.93703	98.78911	97.27138
2383.585	94.73487	94.79588	98.75169	97.16787
2385.513	94.55929	94.67838	98.66162	97.12219
2387.442	94.44802	94.58926	98.55652	97.15337
2389.37	94.44226	94.28739	98.46123	97.17009
2391.299	94.25077	93.95997	98.40262	97.1308
2393.227	94.0547	93.67078	98.35028	97.04352
2395.156	93.86073	93.42993	98.29514	97.00296
2397.084	93.76797	93.22462	98.24225	97.05851
2399.012	93.74983	93.01476	98.21055	97.14055
2400.941	93.74911	92.72787	98.19475	97.14946

2402.869	93.83254	92.37759	98.1838	97.12241
2404.798	93.9085	92.12836	98.18122	97.12635
2406.726	93.88697	92.00294	98.17664	97.12963
2408.655	93.76836	91.90374	98.13481	97.11466
2410.583	93.53492	91.78226	98.06448	97.09955
2412.512	93.32044	91.67174	97.98698	97.06124
2414.44	93.13039	91.59485	97.91362	97.00883
2416.369	93.05144	91.51799	97.84832	96.98988
2418.297	92.9938	91.44444	97.80975	97.00446
2420.226	92.89304	91.36227	97.78561	97.01871
2422.154	92.73466	91.31123	97.75228	97.0344
2424.083	92.60394	91.2705	97.7225	97.06575
2426.011	92.59277	91.18716	97.71179	97.08632
2427.939	92.59525	91.09209	97.70377	97.0383
2429.868	92.6638	90.9969	97.68207	96.9136
2431.796	92.73045	90.89574	97.65097	96.7733
2433.725	92.69396	90.77023	97.61623	96.69785
2435.653	92.61526	90.71446	97.59032	96.69651
2437.582	92.54314	90.69754	97.5875	96.70971
2439.51	92.48723	90.60509	97.6001	96.68389
2441.439	92.42786	90.49314	97.59712	96.63983
2443.367	92.39333	90.38931	97.56316	96.61258
2445.296	92.36761	90.23811	97.50328	96.55967
2447.224	92.28682	90.10767	97.42965	96.4788
2449.153	92.19688	90.01092	97.36702	96.41221
2451.081	92.16306	90.0122	97.32279	96.3839
2453.01	92.10607	89.96585	97.29219	96.39928
2454.938	91.95926	89.86478	97.27971	96.41267
2456.866	91.75433	89.8399	97.2817	96.38614
2458.795	91.61414	89.79556	97.27255	96.34716
2460.723	91.53419	89.71473	97.24915	96.33699
2462.652	91.50095	89.64349	97.24397	96.30338
2464.58	91.39362	89.55649	97.26252	96.19705
2466.509	91.27291	89.47846	97.26582	96.06791
2468.437	91.17739	89.3961	97.22666	95.98972
2470.366	91.05148	89.26094	97.16465	95.98272
2472.294	91.0011	89.11496	97.12427	96.00166
2474.223	90.97432	88.93725	97.1088	95.97599
2476.151	90.93715	88.70303	97.07905	95.89504
2478.08	90.84675	88.4831	97.01465	95.79329
2480.008	90.71275	88.24154	96.93261	95.71634
2481.937	90.59806	88.00197	96.85121	95.67987
2483.865	90.49224	87.77504	96.76338	95.66252

2485.793	90.37174	87.57265	96.66641	95.67286
2487.722	90.25137	87.44243	96.6039	95.69524
2489.65	90.13938	87.29243	96.60318	95.70024
2491.579	90.08463	87.10033	96.62646	95.67589
2493.507	90.0715	86.96522	96.62627	95.62289
2495.436	90.20277	86.84379	96.61359	95.54711
2497.364	90.37231	86.70998	96.62553	95.48071
2499.293	90.41063	86.50811	96.66526	95.46322
2501.221	90.37572	86.28326	96.71134	95.47827
2503.15	90.38297	86.04286	96.74609	95.49419
2505.078	90.45197	85.83071	96.75819	95.50594
2507.007	90.45295	85.69271	96.75009	95.49871
2508.935	90.32306	85.58257	96.72637	95.44771
2510.864	90.10229	85.4889	96.69468	95.36797
2512.792	89.90376	85.41428	96.66479	95.32806
2514.72	89.76819	85.27888	96.62595	95.36953
2516.649	89.6579	85.08266	96.57732	95.40921
2518.577	89.60007	84.89326	96.52939	95.41674
2520.506	89.57567	84.71336	96.49044	95.446
2522.434	89.55525	84.57529	96.44019	95.53171
2524.363	89.42872	84.45754	96.38852	95.61804
2526.291	89.25572	84.2974	96.36295	95.66348
2528.22	89.13088	84.13341	96.34445	95.684
2530.148	89.05814	83.95061	96.30069	95.66953
2532.077	89.00662	83.8533	96.24109	95.56046
2534.005	88.94757	83.8306	96.20903	95.34399
2535.934	88.94836	83.72202	96.21351	95.13618
2537.862	88.97746	83.54984	96.21426	95.01042
2539.791	88.97924	83.44381	96.18558	94.89516
2541.719	89.00818	83.32735	96.13863	94.72102
2543.647	88.97742	83.13066	96.07859	94.51264
2545.576	88.89072	82.93638	96.00548	94.34056
2547.504	88.82552	82.77981	95.92128	94.25965
2549.433	88.73016	82.64795	95.84879	94.29932
2551.361	88.64595	82.57008	95.80061	94.38609
2553.29	88.51276	82.50697	95.77019	94.39665
2555.218	88.22822	82.47507	95.75132	94.27703
2557.147	87.99796	82.46414	95.74423	94.15831
2559.075	87.90913	82.44345	95.74898	94.11961
2561.004	87.83375	82.3631	95.73126	94.15012
2562.932	87.74609	82.2835	95.69349	94.2023
2564.861	87.5987	82.21269	95.65691	94.23677
2566.789	87.42817	82.13362	95.64673	94.20226

2568.718	87.33345	82.04418	95.66603	94.11727
2570.646	87.22746	81.92706	95.69004	94.01293
2572.574	87.12078	81.7554	95.70076	93.90934
2574.503	87.01144	81.56226	95.68993	93.85124
2576.431	86.91636	81.367	95.65613	93.87471
2578.36	86.80505	81.25423	95.60565	93.9213
2580.288	86.63596	81.14534	95.54023	93.92494
2582.217	86.41662	80.96679	95.45188	93.90182
2584.145	86.17851	80.79992	95.35033	93.95302
2586.074	86.01416	80.71313	95.26081	94.08987
2588.002	85.86267	80.55028	95.19963	94.17955
2589.931	85.71313	80.30654	95.16205	94.11615
2591.859	85.63421	80.05286	95.14018	93.9509
2593.788	85.56641	79.7804	95.11515	93.77307
2595.716	85.42692	79.47713	95.07095	93.63168
2597.645	85.27114	79.16142	95.00275	93.51326
2599.573	85.0865	78.90497	94.92154	93.40752
2601.501	84.87246	78.68388	94.85941	93.29907
2603.43	84.81034	78.38907	94.82877	93.15827
2605.358	84.78559	78.05132	94.81717	93.00081
2607.287	84.7226	77.70025	94.78788	92.90211
2609.215	84.67389	77.39537	94.72293	92.91013
2611.144	84.53438	77.12745	94.63779	92.98732
2613.072	84.46833	76.87921	94.539	93.07679
2615.001	84.54916	76.65889	94.43683	93.1364
2616.929	84.59492	76.42599	94.34222	93.15289
2618.858	84.59332	76.16441	94.26003	93.16715
2620.786	84.59994	75.90642	94.18524	93.214
2622.715	84.68184	75.66412	94.13487	93.2537
2624.643	84.74375	75.49035	94.11348	93.20098
2626.572	84.77224	75.29314	94.11482	93.05547
2628.5	84.83133	75.04102	94.12453	92.91335
2630.428	84.89579	74.80893	94.11976	92.81936
2632.357	84.92613	74.56983	94.09861	92.73821
2634.285	84.90273	74.27652	94.08198	92.6603
2636.214	84.89993	73.98115	94.08094	92.62685
2638.142	84.84515	73.68796	94.094	92.63635
2640.071	84.68392	73.43528	94.11832	92.63827
2641.999	84.51688	73.18106	94.13256	92.61116
2643.928	84.34055	72.92587	94.12087	92.59048
2645.856	84.17331	72.7768	94.08759	92.61655
2647.785	84.06358	72.67356	94.03911	92.68111
2649.713	83.90735	72.47976	93.98013	92.73129

2651.642	83.62904	72.30173	93.90883	92.72595
2653.57	83.4509	72.22144	93.84111	92.69315
2655.499	83.26713	72.20092	93.78506	92.71501
2657.427	82.99477	72.1954	93.74991	92.78959
2659.355	82.93704	72.11617	93.7333	92.83062
2661.284	82.98066	71.99937	93.71555	92.79893
2663.212	82.90292	71.92644	93.6622	92.75444
2665.141	82.75497	71.88854	93.59099	92.72253
2667.069	82.49583	71.82302	93.53833	92.70468
2668.998	82.25449	71.71651	93.50442	92.70488
2670.926	82.06782	71.62242	93.4704	92.71112
2672.855	81.94441	71.48929	93.42727	92.70202
2674.783	81.82565	71.42072	93.36856	92.68443
2676.712	81.72635	71.44284	93.30395	92.65697
2678.64	81.69756	71.41277	93.25309	92.58609
2680.569	81.58479	71.2662	93.20877	92.48561
2682.497	81.36558	71.04179	93.1558	92.36963
2684.426	81.14194	70.78049	93.09923	92.22837
2686.354	80.9904	70.46716	93.03651	92.0419
2688.282	80.81612	70.12672	92.98289	91.85423
2690.211	80.60724	69.85081	92.95081	91.72743
2692.139	80.46913	69.6368	92.92727	91.69099
2694.068	80.38999	69.39612	92.87959	91.72494
2695.996	80.25188	69.09753	92.81595	91.75168
2697.925	80.12709	68.79055	92.75669	91.74032
2699.853	80.09755	68.51165	92.70173	91.69838
2701.782	79.96198	68.23565	92.65144	91.64876
2703.71	79.78677	67.96019	92.62147	91.60085
2705.639	79.71263	67.66747	92.61085	91.61994
2707.567	79.53932	67.4304	92.59874	91.70205
2709.496	79.20541	67.30318	92.55731	91.74605
2711.424	78.91187	67.17697	92.48567	91.71875
2713.353	78.6319	66.94882	92.39896	91.69458
2715.281	78.34163	66.66355	92.3145	91.71618
2717.209	78.13256	66.43076	92.22638	91.74825
2719.138	78.0601	66.27142	92.13077	91.72913
2721.066	77.96252	66.14125	92.0397	91.65746
2722.995	77.8924	65.91241	91.96619	91.56902
2724.923	77.77397	65.56175	91.89828	91.47122
2726.852	77.48283	65.14584	91.83375	91.35778
2728.78	77.2207	64.71654	91.7726	91.26471
2730.709	77.09109	64.27258	91.68545	91.18627
2732.637	76.86361	63.8148	91.57075	91.07712

2734.566	76.51938	63.42465	91.48361	90.90483
2736.494	76.30521	63.12019	91.46296	90.71475
2738.423	76.09171	62.81355	91.46667	90.5461
2740.351	75.74023	62.43548	91.44423	90.41413
2742.28	75.36163	62.025	91.4066	90.30155
2744.208	75.04398	61.67383	91.37903	90.20776
2746.136	74.83484	61.41251	91.35616	90.17556
2748.065	74.69543	61.12614	91.29869	90.21309
2749.993	74.59924	60.82733	91.21537	90.2843
2751.922	74.56541	60.51297	91.12185	90.32297
2753.85	74.51657	60.12042	91.02449	90.32565
2755.779	74.46585	59.72963	90.9345	90.31042
2757.707	74.41375	59.29961	90.88937	90.29318
2759.636	74.18792	58.82571	90.88317	90.32063
2761.564	73.98727	58.40206	90.85832	90.37434
2763.493	74.00854	58.04035	90.79108	90.34566
2765.421	74.08088	57.70864	90.70376	90.22502
2767.35	74.033	57.36429	90.63118	90.11998
2769.278	73.94305	56.99045	90.56678	90.07579
2771.207	73.80677	56.63771	90.50614	90.02287
2773.135	73.5659	56.25313	90.44937	89.90908
2775.063	73.27517	55.83821	90.38014	89.73918
2776.992	72.99687	55.42942	90.26694	89.54069
2778.92	72.64048	55.058	90.13713	89.35032
2780.849	72.2862	54.67045	90.04474	89.23393
2782.777	71.99571	54.29122	89.97396	89.20055
2784.706	71.69445	53.89737	89.87737	89.21024
2786.634	71.32768	53.45285	89.76326	89.20987
2788.563	71.11443	53.10681	89.67336	89.14386
2790.491	71.05281	52.8518	89.58984	89.00448
2792.42	71.00088	52.53594	89.48728	88.87838
2794.348	70.88642	52.16052	89.38544	88.78911
2796.277	70.74406	51.77886	89.31407	88.66366
2798.205	70.60905	51.36868	89.24039	88.50423
2800.134	70.44501	50.92068	89.12862	88.36384
2802.062	70.29302	50.54962	89.00414	88.22106
2803.99	70.16615	50.2489	88.90208	88.09725
2805.919	69.96396	49.96124	88.83708	88.05747
2807.847	69.83732	49.63503	88.78513	88.05971
2809.776	69.81596	49.29757	88.71908	88.0118
2811.704	69.66384	49.00364	88.62118	87.91057
2813.633	69.34895	48.79931	88.50535	87.79019
2815.561	68.99499	48.56166	88.39208	87.69432

2817.49	68.62687	48.25103	88.31134	87.68275
2819.418	68.20578	47.89257	88.25729	87.7667
2821.347	67.8038	47.54447	88.18282	87.83813
2823.275	67.48437	47.17164	88.05853	87.86267
2825.204	67.12801	46.77031	87.93179	87.90756
2827.132	66.75458	46.35144	87.85032	87.98639
2829.061	66.37092	46.05548	87.77373	88.04437
2830.989	65.92349	45.80888	87.63112	88.02952
2832.917	65.34589	45.47078	87.42295	87.93534
2834.846	64.82251	45.16539	87.19133	87.77258
2836.774	64.31054	44.83027	86.93329	87.57888
2838.703	63.82556	44.43646	86.65253	87.34158
2840.631	63.40067	43.99675	86.40667	87.04519
2842.56	63.05048	43.48737	86.23005	86.71466
2844.488	62.74505	42.98072	86.09007	86.38943
2846.417	62.47775	42.48321	85.9522	86.07623
2848.345	62.14151	41.94302	85.82171	85.80553
2850.274	61.69167	41.31713	85.70986	85.53874
2852.202	61.13346	40.57411	85.61408	85.22293
2854.131	60.57445	39.77237	85.52135	84.89724
2856.059	60.06403	39.0009	85.4326	84.66274
2857.988	59.464	38.22259	85.34589	84.52913
2859.916	58.82501	37.47434	85.25721	84.44582
2861.844	58.24612	36.74109	85.16349	84.3963
2863.773	57.73254	36.06723	85.105	84.39847
2865.701	57.34806	35.46414	85.10335	84.44127
2867.63	57.00093	34.8947	85.10326	84.47941
2869.558	56.62018	34.33802	85.03174	84.44214
2871.487	56.24887	33.96007	84.91146	84.35253
2873.415	55.91058	33.725	84.79895	84.26004
2875.344	55.61643	33.52317	84.71492	84.19171
2877.272	55.33096	33.34238	84.66613	84.11318
2879.201	55.11106	33.23877	84.65242	84.02165
2881.129	55.20076	33.23849	84.63701	83.93185
2883.058	55.48531	33.23935	84.59656	83.84666
2884.986	55.82482	33.31092	84.55554	83.75139
2886.915	56.10672	33.43036	84.53399	83.65093
2888.843	56.39488	33.53107	84.51532	83.54887
2890.771	56.6878	33.53974	84.47565	83.4336
2892.7	56.89705	33.48293	84.4112	83.30461
2894.628	56.85308	33.36256	84.33497	83.23187
2896.557	56.6846	33.18446	84.23481	83.23843
2898.485	56.44664	32.94391	84.077	83.2381

2900.414	56.0666	32.63602	83.84918	83.14639
2902.342	55.61603	32.30717	83.58993	82.97726
2904.271	55.07536	31.8813	83.33373	82.74208
2906.199	54.47999	31.37394	83.07169	82.41333
2908.128	53.85731	30.86176	82.78536	81.99382
2910.056	53.3047	30.33902	82.49432	81.58271
2911.985	52.76614	29.72891	82.22284	81.26505
2913.913	52.03163	29.00854	81.96946	81.00849
2915.842	51.2646	28.27052	81.73228	80.71236
2917.77	50.62033	27.7072	81.51218	80.38228
2919.698	50.10584	27.12059	81.34383	80.10398
2921.627	49.63319	26.54571	81.25518	79.88784
2923.555	49.15578	26.0852	81.23063	79.73769
2925.484	48.82118	25.6278	81.23456	79.72637
2927.412	48.66314	25.16725	81.27591	79.88387
2929.341	48.49813	24.7727	81.39238	80.08425
2931.269	48.3933	24.50037	81.58471	80.23319
2933.198	48.40814	24.21583	81.80677	80.41566
2935.126	48.4389	23.86493	81.99323	80.72194
2937.055	48.38655	23.61393	82.12353	81.07095
2938.983	48.21401	23.53978	82.213	81.34036
2940.912	47.9883	23.46905	82.2558	81.48181
2942.84	47.87521	23.43644	82.25276	81.50227
2944.769	47.78241	23.47519	82.22667	81.43747
2946.697	47.62478	23.5876	82.18453	81.36619
2948.625	47.40717	23.71142	82.10136	81.33644
2950.554	47.1916	23.74114	81.9948	81.3423
2952.482	47.06507	23.7181	81.90128	81.34015
2954.411	46.98793	23.81886	81.83451	81.30041
2956.339	46.93657	23.99271	81.76329	81.23424
2958.268	46.89541	24.0698	81.69589	81.1752
2960.196	46.93018	24.09128	81.6514	81.13994
2962.125	47.11919	24.25029	81.62866	81.09848
2964.053	47.47022	24.45281	81.62859	81.05921
2965.982	47.82914	24.57733	81.69186	81.03976
2967.91	48.07307	24.73708	81.86048	81.03969
2969.839	48.39298	24.8797	82.12007	81.09286
2971.767	48.83381	24.96055	82.40642	81.28125
2973.696	49.30349	25.09869	82.67071	81.59724
2975.624	49.85658	25.28278	82.92845	81.88492
2977.552	50.49235	25.47361	83.19203	82.06425
2979.481	51.18119	25.55617	83.41408	82.19588
2981.409	51.78505	25.60217	83.57177	82.37695

2983.338	52.24685	25.76718	83.70265	82.63922
2985.266	52.74491	25.9348	83.83342	82.95366
2987.195	53.30035	26.02572	83.95878	83.25256
2989.123	53.75957	26.24061	84.08906	83.51465
2991.052	54.03296	26.48175	84.20697	83.75736
2992.98	54.14311	26.64673	84.254	83.94028
2994.909	54.21288	26.68986	84.22221	84.02629
2996.837	54.39487	26.74009	84.17484	84.02038
2998.766	54.60576	26.84096	84.15967	83.9366
3000.694	54.78838	26.92368	84.14261	83.75306
3002.623	54.86144	26.92285	84.10983	83.54686
3004.551	54.89075	26.82764	84.11285	83.33831
3006.479	54.93404	26.75228	84.17667	83.07858
3008.408	54.86653	26.76255	84.25396	82.77635
3010.336	54.665	26.7132	84.27923	82.5377
3012.265	54.31604	26.7115	84.23742	82.40549
3014.193	53.96479	26.79559	84.15	82.30966
3016.122	53.73318	26.79021	84.04553	82.22915
3018.05	53.39309	26.73084	83.92584	82.18602
3019.979	53.05728	26.68649	83.77299	82.17148
3021.907	52.86698	26.58158	83.61622	82.16224
3023.836	52.66058	26.45966	83.50325	82.163
3025.764	52.47973	26.28787	83.42064	82.19171
3027.693	52.2635	26.11554	83.33346	82.23043
3029.621	51.90185	26.00374	83.23428	82.27426
3031.55	51.52086	25.81751	83.127	82.27903
3033.478	51.0512	25.51783	83.01317	82.20237
3035.406	50.51636	25.24622	82.91105	82.02012
3037.335	50.03918	24.96289	82.8463	81.77231
3039.263	49.6442	24.6214	82.79391	81.55181
3041.192	49.3442	24.2717	82.75521	81.38773
3043.12	49.12547	24.00243	82.74837	81.20736
3045.049	48.85827	23.72548	82.74717	80.97651
3046.977	48.57356	23.31717	82.70044	80.80905
3048.906	48.25757	22.90338	82.61273	80.78168
3050.834	48.012	22.56397	82.51183	80.80237
3052.763	47.74561	22.30204	82.41263	80.70319
3054.691	47.43842	22.10131	82.33267	80.47313
3056.62	47.21294	21.92062	82.24036	80.21851
3058.548	46.95706	21.80272	82.08705	79.98445
3060.477	46.75178	21.65915	81.91648	79.76435
3062.405	46.68729	21.4059	81.79763	79.5975
3064.333	46.59536	21.12644	81.72626	79.51557

3066.262	46.63691	20.84488	81.66925	79.50702
3068.19	46.81927	20.69055	81.611	79.54784
3070.119	46.98693	20.68048	81.53978	79.59953
3072.047	47.17287	20.62761	81.45748	79.62598
3073.976	47.34387	20.48143	81.40619	79.65611
3075.904	47.41919	20.34448	81.41182	79.72604
3077.833	47.52056	20.23376	81.44749	79.84793
3079.761	47.60111	20.18497	81.48179	79.98547
3081.69	47.59947	20.23442	81.51745	80.14449
3083.618	47.62842	20.31088	81.57392	80.30115
3085.547	47.65451	20.33725	81.65954	80.43112
3087.475	47.60195	20.38657	81.78901	80.50295
3089.404	47.46562	20.49363	81.93698	80.54178
3091.332	47.25804	20.55126	82.05856	80.56392
3093.26	47.11642	20.5556	82.13946	80.57107
3095.189	47.04633	20.66054	82.19048	80.53943
3097.117	47.00278	20.83697	82.22119	80.46285
3099.046	46.94358	20.98306	82.24291	80.37566
3100.974	46.77476	21.10435	82.24927	80.34462
3102.903	46.57697	21.24988	82.23332	80.37027
3104.831	46.35699	21.35328	82.19965	80.40105
3106.76	46.23844	21.36753	82.17485	80.35401
3108.688	46.28388	21.41648	82.1567	80.22999
3110.617	46.26833	21.4507	82.13831	80.06606
3112.545	46.32589	21.54608	82.11535	79.85806
3114.474	46.47038	21.73089	82.08291	79.57677
3116.402	46.48523	21.85713	82.01906	79.28063
3118.331	46.40658	21.86228	81.93201	79.04679
3120.259	46.30719	21.80387	81.8391	78.88914
3122.188	46.21607	21.73302	81.74256	78.80739
3124.116	46.16179	21.75309	81.6405	78.82182
3126.044	46.14695	21.78665	81.53579	78.92766
3127.973	46.09303	21.75572	81.39246	79.07752
3129.901	46.00328	21.65107	81.20355	79.19544
3131.83	45.80434	21.41762	81.02855	79.24569
3133.758	45.60167	21.18205	80.89659	79.24349
3135.687	45.43002	20.97877	80.76073	79.17651
3137.615	45.13242	20.71334	80.59013	79.08242
3139.544	44.88175	20.45507	80.41366	79.07818
3141.472	44.65133	20.20773	80.28758	79.18797
3143.401	44.28191	19.94878	80.23659	79.2424
3145.329	43.87881	19.73512	80.24162	79.15726
3147.258	43.40055	19.51019	80.25857	79.02694

3149.186	43.00796	19.26472	80.26319	78.9362
3151.115	42.64958	19.02281	80.24335	78.84533
3153.043	42.29926	18.88987	80.19769	78.73982
3154.971	41.95812	18.92186	80.15174	78.59917
3156.9	41.56901	18.9697	80.12747	78.40753
3158.828	41.27833	19.01285	80.08022	78.22643
3160.757	40.90527	18.94342	79.97774	78.161
3162.685	40.49425	18.8264	79.85721	78.12943
3164.614	40.27333	18.66249	79.75556	77.97109
3166.542	40.14366	18.4966	79.64855	77.68724
3168.471	40.07571	18.3547	79.54345	77.35872
3170.399	40.0534	18.25405	79.45936	76.9681
3172.328	40.08745	18.16405	79.36805	76.5035
3174.256	39.96664	17.95517	79.23006	76.06873
3176.185	39.78223	17.67937	79.07392	75.80266
3178.113	39.65725	17.32389	78.95332	75.66205
3180.042	39.51299	17.03111	78.90607	75.53754
3181.97	39.23731	16.78729	78.91964	75.40213
3183.898	38.83043	16.45813	78.95981	75.33283
3185.827	38.51909	16.1488	78.99177	75.34747
3187.755	38.18381	15.85793	78.97186	75.46503
3189.684	37.84157	15.62155	78.88337	75.63073
3191.612	37.53183	15.39985	78.77494	75.75718
3193.541	37.18327	15.09058	78.68713	75.79305
3195.469	36.83232	14.85176	78.59633	75.801
3197.398	36.4456	14.76238	78.48714	75.8348
3199.326	36.2163	14.7186	78.38008	75.85076
3201.255	36.05631	14.64721	78.28389	75.74413
3203.183	35.94118	14.42574	78.18473	75.4869
3205.112	35.84409	14.16528	78.08798	75.16469
3207.04	35.70328	13.85118	77.98951	74.82534
3208.969	35.69938	13.63339	77.8679	74.47364
3210.897	35.79379	13.54737	77.71614	74.19841
3212.825	35.83209	13.44227	77.55286	74.05361
3214.754	35.77946	13.30488	77.40633	73.9273
3216.682	35.6758	13.224	77.31167	73.7571
3218.611	35.54336	13.14812	77.30177	73.65177
3220.539	35.28997	12.95366	77.3325	73.66772
3222.468	35.17799	12.81235	77.32933	73.679
3224.396	35.13986	12.70217	77.27856	73.65667
3226.325	34.98369	12.62562	77.21469	73.70476
3228.253	34.74564	12.57749	77.14443	73.79334
3230.182	34.52803	12.57342	77.09741	73.80724

3232.11	34.34068	12.68879	77.09696	73.76446
3234.039	34.12009	12.77401	77.14873	73.75425
3235.967	33.96376	12.81477	77.22307	73.76054
3237.896	33.84443	12.81736	77.28733	73.75435
3239.824	33.69728	12.70779	77.31172	73.72214
3241.752	33.48402	12.61483	77.30388	73.6211
3243.681	33.35209	12.49288	77.30188	73.48474
3245.609	33.47992	12.23892	77.3059	73.4197
3247.538	33.50772	11.96626	77.29869	73.42373
3249.466	33.28137	11.66381	77.25703	73.36728
3251.395	32.95831	11.38874	77.17554	73.20845
3253.323	32.68751	11.06346	77.08781	72.97657
3255.252	32.56642	10.73574	77.03127	72.717
3257.18	32.4803	10.54774	76.99008	72.49356
3259.109	32.32692	10.37737	76.9067	72.3864
3261.037	32.09232	10.18303	76.78898	72.37897
3262.966	31.77871	10.00277	76.70116	72.40109
3264.894	31.50031	9.70339	76.66378	72.41486
3266.823	31.36491	9.47164	76.64553	72.41832
3268.751	31.38552	9.41601	76.64585	72.42474
3270.679	31.24284	9.324	76.66799	72.49599
3272.608	30.9689	9.23935	76.6883	72.65744
3274.536	30.66941	9.22458	76.65835	72.81537
3276.465	30.40655	9.29966	76.57307	72.90447
3278.393	30.21229	9.4376	76.4589	72.91176
3280.322	30.08256	9.47105	76.3388	72.88073
3282.25	30.12542	9.40834	76.22963	72.86246
3284.179	30.1157	9.34632	76.13957	72.85133
3286.107	29.90925	9.35031	76.06578	72.76064
3288.036	29.73245	9.46756	75.99175	72.63676
3289.964	29.75911	9.56487	75.92263	72.59533
3291.893	29.75572	9.53582	75.87939	72.56952
3293.821	29.50211	9.5073	75.85915	72.43974
3295.75	29.17406	9.42411	75.84945	72.32663
3297.678	29.04317	9.34536	75.84611	72.35007
3299.606	29.07056	9.36297	75.86863	72.46536
3301.535	29.12524	9.47889	75.92149	72.54565
3303.463	29.11505	9.69599	75.98307	72.60026
3305.392	29.17044	9.8941	76.0433	72.63125
3307.32	29.27874	9.96303	76.11012	72.64369
3309.249	29.30535	9.98585	76.17725	72.66487
3311.177	29.46238	9.98706	76.22732	72.71699
3313.106	29.72594	10.04016	76.22193	72.77253

3315.034	29.91707	10.07977	76.15512	72.81116
3316.963	29.94453	10.06029	76.06794	72.85108
3318.891	29.97371	10.06462	75.99827	72.91701
3320.82	29.91147	10.08576	75.93319	72.93594
3322.748	29.88154	10.10256	75.86556	72.87195
3324.677	30.05748	10.06307	75.81051	72.78262
3326.605	30.24622	9.84911	75.74852	72.74442
3328.533	30.36994	9.5625	75.65975	72.68456
3330.462	30.33807	9.39964	75.56867	72.48714
3332.39	30.34853	9.28361	75.51293	72.19833
3334.319	30.45456	9.07904	75.47835	71.96889
3336.247	30.4771	8.79616	75.43605	71.90131
3338.176	30.41284	8.51669	75.39284	71.96335
3340.104	30.35488	8.33231	75.38928	72.05391
3342.033	30.36509	8.20603	75.42178	72.14808
3343.961	30.35707	8.15342	75.44996	72.22413
3345.89	30.33251	8.16727	75.47221	72.25798
3347.818	30.31277	8.24343	75.52251	72.2452
3349.747	30.36949	8.35656	75.58966	72.23802
3351.675	30.60704	8.52476	75.62703	72.24682
3353.604	30.82084	8.65745	75.64778	72.19923
3355.532	30.91599	8.73352	75.70095	72.0221
3357.46	30.9648	8.84041	75.7891	71.74376
3359.389	30.91705	9.00919	75.87439	71.49174
3361.317	30.72474	9.21695	75.94854	71.35638
3363.246	30.5393	9.44725	76.00651	71.26792
3365.174	30.52183	9.61299	76.01744	71.15518
3367.103	30.63327	9.72357	75.96458	71.07402
3369.031	30.85168	9.72405	75.88092	71.09957
3370.96	31.03401	9.67141	75.79368	71.24299
3372.888	30.96016	9.496	75.69487	71.44806
3374.817	30.78134	9.30316	75.59401	71.75649
3376.745	30.75161	9.21185	75.51355	72.15981
3378.674	30.83178	9.05722	75.44022	72.5461
3380.602	30.86229	8.85546	75.34135	72.81086
3382.531	30.83783	8.66377	75.22679	72.89002
3384.459	30.87521	8.49114	75.16023	72.76095
3386.387	30.95796	8.45633	75.15126	72.53797
3388.316	30.92034	8.48863	75.15239	72.42174
3390.244	30.80302	8.43646	75.13816	72.4098
3392.173	30.94832	8.40303	75.12662	72.32788
3394.101	31.15972	8.57981	75.13688	72.16853
3396.03	31.27676	8.87339	75.1517	72.09159

3397.958	31.41015	9.10467	75.17702	72.20669
3399.887	31.35807	9.23687	75.22404	72.38373
3401.815	31.14733	9.34033	75.25848	72.43856
3403.744	30.90925	9.50498	75.24122	72.37666
3405.672	30.83102	9.64293	75.17668	72.3221
3407.601	31.08821	9.76973	75.1105	72.29391
3409.529	31.58721	9.92557	75.04234	72.23401
3411.458	32.00634	10.07153	74.98797	72.12768
3413.386	32.25119	10.15759	74.96782	72.03463
3415.314	32.33545	10.19638	74.95276	71.92108
3417.243	32.30355	10.21962	74.9069	71.72741
3419.171	32.09855	10.20852	74.89513	71.45165
3421.1	31.92588	10.28165	74.96831	71.1777
3423.028	31.91155	10.41711	75.07085	70.98202
3424.957	31.85052	10.43772	75.12866	70.9648
3426.885	31.77216	10.47827	75.15699	71.12513
3428.814	31.73195	10.64512	75.1902	71.33293
3430.742	31.84779	10.90856	75.23816	71.46705
3432.671	32.06725	11.23809	75.28046	71.60233
3434.599	32.35072	11.48817	75.30734	71.81889
3436.528	32.64704	11.72219	75.34967	72.09475
3438.456	32.85909	12.01409	75.4116	72.33412
3440.385	32.88838	12.37661	75.43754	72.47655
3442.313	32.86077	12.62602	75.43605	72.53675
3444.241	32.85959	12.70885	75.44424	72.59717
3446.17	32.8624	12.67602	75.43925	72.66749
3448.098	33.08161	12.59396	75.3778	72.73651
3450.027	33.50973	12.52576	75.30793	72.77516
3451.955	34.07457	12.40825	75.28488	72.80378
3453.884	34.59186	12.2123	75.31326	72.86616
3455.812	34.7965	12.02704	75.38031	73.07283
3457.741	34.73761	11.80806	75.49199	73.37838
3459.669	34.65085	11.58695	75.61414	73.68083
3461.598	34.56656	11.49938	75.69317	73.94962
3463.526	34.44247	11.58754	75.7341	74.18968
3465.455	34.25683	11.74713	75.77855	74.35634
3467.383	34.15265	11.80752	75.83251	74.47815
3469.312	34.11802	11.81765	75.88107	74.60478
3471.24	34.16237	11.88581	75.91985	74.76219
3473.168	34.2919	12.11066	75.95775	74.89411
3475.097	34.53114	12.35776	75.99789	74.97803
3477.025	34.85041	12.50814	76.02883	74.99435
3478.954	35.06192	12.51525	76.02533	74.92966

3480.882	35.18483	12.53511	76.03222	74.79711
3482.811	35.17246	12.67666	76.04305	74.68669
3484.739	35.08692	12.80257	75.99002	74.58039
3486.668	35.15838	12.90321	75.8655	74.38917
3488.596	35.39616	12.95357	75.77761	74.10301
3490.525	35.58003	12.93829	75.79942	73.82657
3492.453	35.84921	12.87956	75.89858	73.67542
3494.382	36.23347	12.87466	76.0266	73.69569
3496.31	36.51262	12.96913	76.1533	73.77176
3498.239	36.60192	13.02489	76.28481	73.79966
3500.167	36.5705	13.18058	76.42395	73.81712
3502.095	36.53915	13.45323	76.57866	73.85385
3504.024	36.57204	13.67307	76.74746	73.92556
3505.952	36.71421	14.01129	76.92685	74.09685
3507.881	36.91307	14.37705	77.09819	74.40933
3509.809	37.16582	14.70129	77.23677	74.78049
3511.738	37.52877	14.96419	77.36378	75.14558
3513.666	37.91668	15.14257	77.50556	75.4989
3515.595	38.26336	15.33009	77.63215	75.77075
3517.523	38.55079	15.56587	77.69407	75.95879
3519.452	38.79767	15.70118	77.71446	76.17287
3521.38	39.00192	15.60198	77.69232	76.4537
3523.309	39.17857	15.50213	77.62176	76.57529
3525.237	39.36354	15.41512	77.57654	76.49432
3527.166	39.61134	15.41655	77.63875	76.44596
3529.094	39.82734	15.50806	77.76404	76.59167
3531.022	39.94617	15.57066	77.882	76.88865
3532.951	40.07243	15.68864	77.99635	77.24227
3534.879	40.21716	15.87675	78.10903	77.55482
3536.808	40.32693	16.04264	78.22798	77.75297
3538.736	40.53636	16.20174	78.36349	77.80034
3540.665	40.74062	16.40158	78.52595	77.78671
3542.593	40.91948	16.63368	78.68604	77.84013
3544.522	41.07662	16.83103	78.82963	78.02754
3546.45	41.37141	17.14233	78.94846	78.24839
3548.379	41.74328	17.44542	79.07617	78.38443
3550.307	42.14059	17.79477	79.24665	78.45029
3552.236	42.59009	18.35231	79.43454	78.48881
3554.164	42.92164	18.94082	79.59796	78.54502
3556.093	43.19253	19.35091	79.7336	78.65496
3558.021	43.60067	19.70739	79.84024	78.81584
3559.949	43.97839	20.08471	79.93892	78.89828
3561.878	44.27303	20.53406	80.07305	78.82899

3563.806	44.73033	21.02828	80.25718	78.86441
3565.735	45.28193	21.34606	80.45412	79.20368
3567.663	45.7249	21.49193	80.64688	79.71952
3569.592	46.17535	21.74316	80.8209	80.16189
3571.52	46.57017	22.01555	80.9548	80.53145
3573.449	46.8483	22.30903	81.04787	80.91272
3575.377	47.11583	22.68861	81.12627	81.29293
3577.306	47.24158	23.0799	81.2079	81.57023
3579.234	47.20174	23.51008	81.31426	81.75076
3581.163	47.3441	23.9632	81.44579	81.81851
3583.091	47.64648	24.41082	81.59987	81.80879
3585.02	48.06413	24.78771	81.74306	81.75599
3586.948	48.65891	25.14798	81.85977	81.72248
3588.876	49.28505	25.54295	81.97112	81.79684
3590.805	49.79449	26.06397	82.11087	81.98303
3592.733	50.36345	26.70563	82.28237	82.11503
3594.662	51.0066	27.37669	82.46419	82.15549
3596.59	51.75511	27.93106	82.64699	82.26821
3598.519	52.38818	28.41869	82.8378	82.53069
3600.447	52.95906	28.91395	83.02953	82.83407
3602.376	53.6409	29.3946	83.2271	83.17221
3604.304	54.3669	30.15168	83.46153	83.55429
3606.233	55.10644	31.03964	83.73463	83.93229
3608.161	55.95196	31.72165	83.99136	84.22794
3610.09	56.89169	32.18516	84.19255	84.51762
3612.018	57.41956	32.55526	84.37239	84.85257
3613.947	57.78296	32.97168	84.55465	85.14213
3615.875	58.2508	33.46496	84.77052	85.25765
3617.803	58.59623	33.94837	85.05185	85.24912
3619.732	58.89043	34.54697	85.36471	85.28691
3621.66	59.19406	35.34681	85.61592	85.52009
3623.589	59.26325	36.2616	85.78585	85.93342
3625.517	59.38796	37.24035	85.95106	86.39711
3627.446	59.78076	38.30139	86.18824	86.82203
3629.374	60.22422	39.30671	86.48214	87.05947
3631.303	60.7688	40.2094	86.82088	87.12361
3633.231	61.36237	41.11845	87.1781	87.28503
3635.16	62.09109	41.84801	87.49307	87.64558
3637.088	62.93402	42.54591	87.71447	87.9848
3639.017	63.77956	43.2719	87.89273	88.1534
3640.945	64.78098	43.98526	88.11881	88.29638
3642.874	65.8085	44.64269	88.42306	88.52486
3644.802	66.62109	45.36462	88.77515	88.77439

3646.73	67.54398	46.25807	89.11405	88.93787
3648.659	68.38273	47.15686	89.38623	89.04934
3650.587	69.09138	47.96469	89.62355	89.19557
3652.516	69.92676	48.83508	89.86881	89.38008
3654.444	70.67161	49.7427	90.16755	89.64544
3656.373	71.15589	50.74431	90.50495	90.04477
3658.301	71.31565	51.72451	90.79542	90.59178
3660.23	71.58659	52.62055	90.9675	91.1884
3662.158	71.8894	53.51361	91.10866	91.79168
3664.087	72.08495	54.5488	91.32835	92.4329
3666.015	72.31716	55.77612	91.62733	93.08791
3667.944	72.6153	57.03014	91.94694	93.5842
3669.872	72.99112	58.18362	92.26215	93.94757
3671.801	73.40818	59.25127	92.51881	94.26138
3673.729	73.87184	60.2827	92.68776	94.48003
3675.657	74.44431	61.15195	92.81518	94.53525
3677.586	75.14746	61.82167	92.97074	94.5764
3679.514	75.97733	62.46273	93.15668	94.72251
3681.443	76.94291	63.02742	93.33081	94.91358
3683.371	77.75046	63.71919	93.45484	94.98163
3685.3	78.38659	64.46107	93.53027	94.8447
3687.228	79.15576	65.10906	93.58742	94.58797
3689.157	79.7132	65.75216	93.68287	94.3155
3691.085	80.12481	66.29879	93.81548	94.16511
3693.014	80.68298	66.72565	93.98442	94.14378
3694.942	81.03643	67.12239	94.18389	94.16855
3696.871	81.36353	67.72017	94.35611	94.18609
3698.799	81.89921	68.48957	94.43717	94.24501
3700.728	82.27547	69.12222	94.49216	94.41961
3702.656	82.47302	69.50954	94.62089	94.71293
3704.584	82.44427	69.71707	94.82986	94.95052
3706.513	82.35928	69.87325	95.05001	94.87561
3708.441	82.36179	70.10767	95.22174	94.54301
3710.37	82.73618	70.62934	95.29873	94.21971
3712.298	83.2393	71.23125	95.30293	94.10963
3714.227	83.64454	71.67805	95.24623	94.3051
3716.155	84.06003	71.95718	95.15356	94.65773
3718.084	84.38802	72.05729	95.04516	94.87075
3720.012	84.63364	71.99904	94.96821	94.8645
3721.941	84.8792	72.21935	94.93631	94.74309
3723.869	85.25027	72.61012	94.92199	94.51858
3725.798	85.6476	73.10705	94.90326	94.26118
3727.726	85.80113	73.38551	94.90134	94.11846

3729.655	85.78596	73.43501	94.97759	94.26712
3731.583	85.83353	73.48685	95.15241	94.60255
3733.511	85.77588	73.68573	95.38625	94.96885
3735.44	85.70705	73.78432	95.59088	95.17144
3737.368	86.03149	73.70409	95.70818	95.21557
3739.297	86.36872	73.69418	95.83863	95.21693
3741.225	86.44217	73.89675	96.10728	95.2512
3743.154	86.68456	74.30048	96.46277	95.26063
3745.082	87.11266	74.58739	96.77565	95.16247
3747.011	87.24999	74.89207	97.00071	95.14941
3748.939	87.1223	75.19485	97.13535	95.45672
3750.868	86.89104	75.43736	97.17247	95.99575
3752.796	86.74237	75.68412	97.12233	96.42386
3754.725	86.85524	76.05	97.05573	96.5096
3756.653	87.10503	76.68958	97.01761	96.44649
3758.582	87.12517	77.26311	97.008	96.54803
3760.51	87.09977	77.49577	96.95722	96.73125
3762.438	87.32246	77.59785	96.82812	96.75691
3764.367	87.45593	77.83818	96.66189	96.68376
3766.295	87.49466	78.34045	96.50752	96.71866
3768.224	87.9232	79.10333	96.40582	96.85371
3770.152	88.44502	79.54333	96.34006	96.98339
3772.081	88.50178	79.71071	96.25802	97.00282
3774.009	88.40292	79.69595	96.14472	96.87238
3775.938	88.52531	79.72536	96.0476	96.72003
3777.866	88.98027	79.8736	96.04327	96.71428
3779.795	89.32816	80.1089	96.1331	96.81767
3781.723	89.65578	80.36182	96.24505	96.96965
3783.652	89.87065	80.6724	96.32564	97.12149
3785.58	89.86491	80.91474	96.37697	97.27229
3787.509	89.98624	81.2767	96.39605	97.37071
3789.437	90.42234	81.64294	96.3955	97.35236
3791.365	90.80675	81.87691	96.4334	97.30307
3793.294	90.842	82.19628	96.54991	97.34877
3795.222	90.76047	82.23438	96.73237	97.45558
3797.151	90.68208	82.02046	96.92712	97.42802
3799.079	90.73203	82.00697	97.07392	97.16638
3801.008	90.85638	82.20238	97.13645	96.84324
3802.936	90.93116	82.49216	97.13706	96.59584
3804.865	90.93253	82.69228	97.1209	96.37551
3806.793	91.04132	82.77937	97.1453	96.0866
3808.722	91.28845	83.05703	97.19882	95.88562
3810.65	91.48591	83.43932	97.20459	95.8479

3812.579	91.74187	83.58397	97.17036	95.90476
3814.507	91.91412	83.68028	97.14881	96.02865
3816.436	91.88762	83.89469	97.15841	96.25852
3818.364	91.74693	84.18687	97.1801	96.52555
3820.292	91.79167	84.44072	97.24131	96.85132
3822.221	91.8651	84.69592	97.37326	97.11536
3824.149	91.89183	84.90612	97.58566	97.22651
3826.078	91.81841	85.24684	97.82982	97.26408
3828.006	91.68305	85.6308	98.07099	97.34585
3829.935	91.44745	85.74792	98.28861	97.35011
3831.863	91.3929	85.7166	98.50584	97.12318
3833.792	91.46436	85.88538	98.67776	96.86223
3835.72	91.33966	86.01247	98.7398	96.76886
3837.649	90.87579	86.06067	98.6577	96.85966
3839.577	90.52141	86.10849	98.49409	96.96316
3841.506	90.44239	85.99318	98.33105	96.92624
3843.434	90.57985	85.93828	98.1618	96.72767
3845.363	90.8682	86.24303	98.01741	96.50109
3847.291	91.13121	86.45837	97.90976	96.34677
3849.219	91.40234	86.4507	97.81086	96.22778
3851.148	91.70054	86.60699	97.66881	96.12409
3853.076	91.97794	86.80366	97.513	96.1152
3855.005	92.20959	86.93565	97.40902	96.16658
3856.933	92.42439	87.06812	97.37772	96.21354
3858.862	92.41849	87.07674	97.40442	96.25833
3860.79	92.37882	87.23846	97.49409	96.23183
3862.719	92.56452	87.55863	97.63151	96.13661
3864.647	92.79853	87.68723	97.79305	96.04537
3866.576	93.05967	87.75285	97.94305	95.96279
3868.504	93.31686	87.87016	98.06971	95.82082
3870.433	93.49866	88.0349	98.17778	95.70645
3872.361	93.67218	88.39415	98.24989	95.78357
3874.29	93.59182	88.64289	98.2701	95.95049
3876.218	93.38513	88.67462	98.23111	96.06336
3878.146	93.25951	88.77791	98.1721	96.09745
3880.075	92.90481	89.00927	98.11392	96.13214
3882.003	92.37124	89.29736	98.10226	96.15957
3883.932	92.09371	89.56403	98.21198	96.07763
3885.86	91.98895	89.93614	98.40415	95.79741
3887.789	91.82458	90.30105	98.53656	95.42803
3889.717	91.71324	90.48946	98.54068	95.12829
3891.646	91.51263	90.69004	98.48972	94.93441
3893.574	91.37375	90.78953	98.44815	94.82227

3895.503	91.2417	90.82304	98.40133	94.78663
3897.431	90.93149	90.98704	98.3454	94.79091
3899.36	90.61198	90.97891	98.33384	94.82613
3901.288	90.41522	91.05472	98.34157	94.91741
3903.217	90.22635	91.22375	98.31253	95.06803
3905.145	90.03791	91.2137	98.22271	95.24954
3907.073	90.3214	91.26334	98.10753	95.54863
3909.002	90.83822	91.36184	97.96703	95.9929
3910.93	91.39396	91.42416	97.80105	96.48273
3912.859	91.8862	91.52177	97.61305	96.93254
3914.787	92.32804	91.63248	97.42256	97.35933
3916.716	92.8992	91.71346	97.28119	97.77462
3918.644	93.57883	91.69463	97.25237	98.07647
3920.573	94.29852	91.6226	97.3573	98.17229
3922.501	95.00745	91.65129	97.50455	98.01977
3924.43	95.44015	91.80602	97.62144	97.67752
3926.358	95.67943	91.99092	97.68042	97.31021
3928.287	95.87168	92.21647	97.7166	97.03736
3930.215	95.89959	92.39975	97.71823	96.91493
3932.144	95.66589	92.6496	97.6854	96.85849
3934.072	95.34669	92.75297	97.65989	96.71795
3936	95.08111	92.5807	97.70181	96.4107
3937.929	94.65658	92.42857	97.81223	96.05052
3939.857	94.32368	92.41516	97.90654	95.7177
3941.786	93.99212	92.44594	97.96448	95.54063
3943.714	93.52082	92.5413	98.00584	95.56983
3945.643	93.23678	92.68098	98.03586	95.82446
3947.571	93.05494	92.95385	98.03851	96.13095
3949.5	92.84483	93.20383	98.0211	96.31524
3951.428	92.69229	93.47637	98.02656	96.38818
3953.357	92.65457	93.74027	98.07106	96.46108
3955.285	92.46822	93.84254	98.14329	96.62371
3957.214	92.1453	93.96701	98.18797	96.73342
3959.142	91.95994	94.01163	98.19993	96.74703
3961.071	92.00525	94.04815	98.23469	96.71301
3962.999	92.25311	94.03493	98.33508	96.79892
3964.927	92.59451	93.98915	98.40651	97.07589
3966.856	93.0042	94.02589	98.34282	97.47341
3968.784	93.34623	93.99227	98.18466	97.77399
3970.713	93.44419	93.92525	98.09705	97.83287
3972.641	93.47982	93.89974	98.13675	97.7602
3974.57	93.38395	94.09441	98.23269	97.65917
3976.498	93.20313	94.51379	98.30012	97.54678

3978.427	93.12438	94.51212	98.33452	97.48681
3980.355	93.05843	94.38606	98.35393	97.5701
3982.284	93.22498	94.68941	98.3808	97.4781
3984.212	93.49642	95.02714	98.404	97.36097
3986.141	93.75208	95.62254	98.42353	97.21872
3988.069	93.18557	96.22014	98.4394	97.05134
3989.998	92.28296	97.02358	98.45159	96.85884
3991.926	91.49729	98.383	98.46012	96.64122
3993.854	91.71724	99.73328	98.46498	96.39846
3995.783	93.16064	99.61362	98.46617	96.13059
3997.711	94.41949	96.44867	98.4637	95.83758
3999.64	95.86446	92.27296	98.45756	95.51946

Table 2 Effect of dopamine concentration on the differential friction effect of wool fibers and the pilling grade of wool knitted fabrics

concentration of DA	pilling grade					DFE				
0.5	4.5	4	4.5	4.5	4	0.078	0.068	0.072	0.074	0.077
1	5	5	5	5	4.5	0.062	0.067	0.07	0.065	0.064
2	4	4	3.5	4	3.5	0.078	0.075	0.071	0.08	0.073
5	3.5	3.5	3.5	3	3	0.09	0.085	0.086	0.083	0.088
10	3	3.5	3	3	3.5	0.101	0.096	0.099	0.105	0.107

Table 3Effect of dopamine concentration on the crimping properties of wool fibers

concentration of DA	crimp rate				crimp recovery rate					
0.5	3.87	3.85	3.81	3.83	3.91	4.02	3.47	3.44	3.38	3.45
1	3.41	3.43	3.39	3.37	3.45	4.82	3.12	3.09	3.05	3.07
2	4.04	3.98	4.08	3.91	3.93	3.71	3.75	3.67	3.65	3.69
5	4.52	4.56	4.48	4.42	4.45	3.49	4.06	3.97	3.95	4.03
10	4.86	4.91	4.85	4.82	4.9	3.13	4.86	4.83	4.76	4.73

Table 4 Effect of dopamine concentration on the top breaking strength of wool knitted fabrics

concentration of DA	top breaking strength					
0.5	1	357.3	365.8	369.4	370.1	372.6
1	2	411.8	416.8	398.6	413.1	420.3
2	3	350.7	351.8	346.2	359.6	354.3
5	4	287.6	302.5	290.3	292.5	293.5
10	5	260.3	257.9	268.7	265.1	262.8

Table 5 r under different concentration of DA

concentration of DA	r of 30℃	r of 40℃	r of 50℃	r of 50℃	r of 60℃
1	1.714	15.1	22.3	23.3	15.4
1.3	3.987	19	26.7	49	47.9
1.5	4.648	23	36.2	53.7	53.8
1.7	5.423	27.8	37.9	89.1	110.2
2	6.075	35.9	47.8	102	134.6
